# Regulation of the cell surface expression of classical and non-classical MHC proteins by the human cytomegalovirus UL40 and rhesus cytomegalovirus Rh67 proteins

**DOI:** 10.1128/jvi.01206-24

**Published:** 2024-08-29

**Authors:** Simon Brackenridge, Nessy John, Wanlin He, Klaus Früh, Persephone Borrow, Andrew McMichael

**Affiliations:** 1 Centre for Immuno-Oncology, Nuffield Department of Medicine, University of Oxford, Oxford, United Kingdom; 2 Vaccine & Gene Therapy Institute, Oregon Health & Science University, Beaverton, Oregon, USA; The University of Arizona, Tucson, Arizona, USA

**Keywords:** major histocompatibility complex, cytomegalovirus

## Abstract

The signal sequences of the human cytomegalovirus (CMV) UL40 protein and its rhesus CMV (RhCMV) counterpart, Rh67, contain a peptide (VMAPRT[L/V][F/I/L/V]L, VL9) that is presented by major histocompatibility complex (MHC) antigen E (MHC-E). The CMV VL9 peptides replace VL9 peptides derived from classical MHC (Ia) signal sequences, which are lost when CMV disrupts antigen processing and presentation and MHC Ia expression. This allows infected cells to maintain MHC-E surface expression and escape killing by Natural Killer cells. We demonstrate that processing of the Rh67 VL9 peptide mirrors that of UL40, despite the lack of sequence conservation between the two proteins. Processing of both VL9 peptides is dependent on cleavage of their signal sequences by the host protease signal peptide peptidase. As previously shown for UL40, up-regulation of MHC-E expression by Rh67 requires only its signal sequence, with sequences upstream of VL9 critical for conferring independence from TAP, the transporter associated with antigen processing. Our results also suggest that the mature UL40 and Rh67 proteins contribute to CMV immune evasion by decreasing surface expression of MHC Ia. Unexpectedly, while the Rh67 VL9 peptide is resistant to the effects of Rh67, UL40 can partially counteract the up-regulation of MHC-E expression mediated by its own VL9 peptide. This suggests differences in the mechanisms by which the two VL9 peptides up-regulate MHC-E, and further work will be required to determine if any such differences have implications for translating a RhCMV-vectored simian immunodeficiency virus (SIV) vaccine to HIV-1 using human CMV as a vector.

**IMPORTANCE:**

The protective immune response induced by a rhesus cytomegalovirus (RhCMV)-vectored simian immunodeficiency virus (SIV) vaccine in rhesus macaques depends on the presence of the viral Rh67 gene in the vaccine. The Rh67 protein contains a peptide that allows the RhCMV-infected cells to maintain expression of major histocompatibility complex (MHC) antigen E at the cell surface. We show that production of this peptide, referred to as “VL9,” mirrors that of the equivalent peptide present in the human cytomegalovirus (CMV) protein UL40, despite the little sequence similarity between the two CMV proteins. We also show that the mature UL40 and Rh67 proteins, which have no previously described function, also contribute to CMV immune evasion by reducing cell surface expression of MHC proteins important for the immune system to detect infected cells. Despite these similarities, our work also reveals possible differences between Rh67 and UL40, and these may have implications for the use of human CMV as the vector for a potential HIV-1 vaccine.

## INTRODUCTION

Cell surface expression of the major histocompatibility (MHC) E protein, known as HLA-E in humans, depends on a nonamer peptide (VMAPRT[L/V][F/I/L/V]L, VL9) present in the signal sequences of many MHC class Ia (MHC Ia) allotypes (1, 2). Presentation of this peptide is dependent on cleavage by both the endoplasmic reticulum (ER) membrane resident protease signal peptide peptidase (SPP) (3, 4) and the proteasome (5), import into the ER lumen by the transporter associated with antigen presentation (TAP), as well as tapasin and the peptide loading complex (PLC) (6).

The presence of HLA-E at the cell surface is monitored by Natural Killer (NK) cells via CD94/NKG2 receptor complexes (7, 8) as a proxy for MHC class I expression. Loss of surface HLA-E results in the loss of signaling through the inhibitory NKG2A/CD94 complex, allowing NK cells to kill HLA-E-deficient cells (9). Cell surface expression of HLA-E is, therefore, often maintained by virally infected cells, in contrast to that of classical MHC class I proteins. For example, evasion of CD8^+^ T cell responses by human CMV (HCMV) involves not only direct targeting of the MHC Ia proteins themselves, including retention in the ER by US10 (10) and targeting for degradation by US2 and US11 (11, 12), but also targeting of accessory factors. US6 blocks peptide transport by TAP (13), while US3 inhibits tapasin (14). Such comprehensive disruption of both MHC Ia expression and components of the PLC would be predicted to limit the availability of the VL9 peptide for HLA-E, resulting in NK cell-mediated killing of HCMV-infected cells. However, the signal sequence of the HCMV UL40 protein contains a VL9 peptide, which can be presented by HLA-E in a TAP-independent manner (15, 16), allowing HCMV-infected cells to maintain surface expression of HLA-E and avoid detection by NK cells.

Immune evasion by rhesus CMV (RhCMV) appears to be highly conserved, and equivalents of the HCMV US2, US3, US6, and US11 proteins have been identified that function by conserved mechanisms: Rh182, Rh184, Rh186, and Rh188, respectively (17). RhCMV also appears to evade NK cells in the same way as HCMV, via the Rh67 protein. Despite otherwise appearing unrelated to UL40, Rh67 also contains the VL9 peptide in its signal sequence (18), and we have previously shown that the Rh67 VL9 peptide can stabilize HLA-E at the cell surface (19). More recently, it has been confirmed that presentation of the Rh67 VL9 peptide does not require TAP (20), confirming that Rh67 is indeed the RhCMV equivalent of UL40.

A RhCMV-vectored vaccine containing the simian immunodeficiency virus (SIV) Gag, Rev/Tat/Nef, Env, and Pol sequences elicits an immune response that enables ~55% of vaccinated rhesus macaques to clear SIV infection early after virus challenge (21, 22). Unusually, the CD8^+^ T cell responses induced by this vaccine are not restricted by classical MHC Ia proteins. Instead, approximately two-thirds of the CD8^+^ T cells are restricted by MHC class II, with the rest restricted by Mamu-E, the rhesus macaque homolog of HLA-E (19). A full understanding of exactly how protection is mediated by this vaccine remains to be elucidated, but it has recently been show to depend on the Mamu-E-restricted responses rather than the MHC class II-restricted responses (23, 24).

Successful development of an HCMV-vectored HIV-1 vaccine will likely depend on a detailed understanding of the similarities and differences in the immune evasion strategies employed by RhCMV and HCMV. Indeed, such an understanding may even allow the required CMV genes to be engineered into a more conventional vaccine vector, thereby avoiding the use of HCMV as a vaccine vector. To this end, we have undertaken a detailed comparison of how the VL9 peptides from UL40 and Rh67 are processed for presentation by HLA-E. Our results reveal that the similarities between UL40 and Rh67 extend beyond their independence from TAP. Processing of both the UL40 and Rh67 VL9 peptides is dependent on cleavage by SPP, and the up-regulation of HLA-E mediated by Rh67 requires only the signal sequence, as was reported previously for UL40 (16). Our results also suggest that the mature Rh67 and UL40 proteins may also contribute to the evasion of the host immune response by decreasing surface MHC class I expression and confirm that Rh67 also contributes to the down-regulation of MHC class I expression in cells infected with RhCMV. Unexpectedly, the mature UL40 and Rh67 proteins are also capable of counteracting the up-regulation of HLA-E mediated by the UL40 signal sequences, but not the Rh67 signal sequence. This suggests that the TAP independence of the UL40 and Rh67 VL9 peptides may be achieved by distinct mechanisms and that there may be differences in the level of MHC-E expression required to evade NK cells in humans and macaques. It will be important to determine if these differences are also observed in infected cells, as this could potentially have implications for the successful induction of HLA-E-restricted CD8^+^ T cells by a HCMV-based HIV-1 vaccine.

## MATERIALS AND METHODS

### Expression plasmids and cloning

The single-chain dimer (SCD) of HLA-E*01:01 and β2-microglobulin was as previously described (19), and the HLA-E*01:03 SCD was made by an identical cloning strategy. The coding sequences of HLA-A*02:01, HLA-A*03:01, HLA-B*57:01 and KIR2DS2*001, UL40 (HCMV strain Toledo), and Rh67 (RhCMV strain 68-1) were purchased as synthetic genes from Eurofins with appropriate restriction sites (HindIII and AgeI) and inserted into pEGFP-N1 (Clontech). The various signal HLA-A*02:01 and HLA-A*03:01 constructs with altered signal sequences were made by inserting the wild-type UL40 or Rh67 signal sequences using HindIII and a BamHI site engineered at the start of the mature HLA coding sequence, then mutating by PCR with appropriate primers. Constructs to express peptides in the cytosol were created by inserting annealed oligonucleotides comprising a Kozak sequence, a start codon, the coding sequence of the peptide, and a stop codon, flanked by the overhangs for HindIII and AgeI, into pEGFP-N1 cut with the same enzymes. For the constructs expressing peptides with a signal sequence, a modified version of the signal sequence of HLA-E [with the final codon for alanine (GCG) replaced by one for proline (CCA) to create an MscI site (TGGCCA)] was first inserted into pEGFP-N1 using HindIII and BamHI. Annealed oligos encoding alanine followed by the desired peptide and a stop codon and with the overhang for AgeI at the 3′ end, were inserted after the signal sequence using the MscI and AgeI restriction sites. The mature coding sequences of UL40 and Rh67 (with FspI and BamHI restriction sites) were also inserted into this vector using the MscI and BamHI sites to create versions with their signal sequences replaced by the HLA-E signal sequence. The SPP expression construct was made by inserting cDNA (amplified by reverse transcription PCR [RT-PCR] from 293T cells) into pcDNA3.1neo (Life Technologies) using NheI and NotI sites included in the PCR primers. The catalytically inactive SPP D219A mutant was created using overlap-extension PCR (25) with KOD Hot Start Polymerase (Merck) and appropriate primers. PCR products were purified using QIAquick spin columns (QIAGEN), and the sequences of the oligonucleotides used are shown in Table S1. The HCV Core-E1 expression plasmid was a kind gift of Andrea Magri (University of Oxford). All plasmids were prepared using QIAprep mini spin columns (QIAGEN), and sequences were verified by Sanger sequencing using an ABI3770.

### Antibody clones

The following anti-HLA antibodies conjugated with allophycocyanin were used for staining: anti-HLA-E (clone 3D12) from BioLegend and anti-HLA-A3 (clone GAP.A3) and anti-HLA (clone W6/32) from Life Technologies. The rhesus anti-Mamu-A*01 antibody conjugated with phycoerythrin (PE) was from the Non-Human Primate Reagent Resource (AB_2819290), and the mouse monoclonal antibody specific for the RhCMV Rh152/151 Fc gamma receptor was as previously described (26). For western blotting, rabbit anti-SPP was from Abcam (ab190253), mouse anti-Hepatitis C virus Core protein (clone C7-50) was from Life Technologies, and mouse anti-β tubulin conjugated with DyLight-680 was from Invitrogen. For the immunofluorescence confocal microscopy experiments, polyclonal rabbit anti-human calnexin (ab22595) and goat anti-rabbit Alexa Fluor 568 (ab17547) secondary antibody were from Abcam.

### Cell culture and transfection

HEK 293T cells were maintained between 10% and 90% confluency at 37°C/5% CO_2_ in Dulbecco's modified Eagle medium (DMEM; Life Technologies) supplemented with 10% fetal bovine serum (FBS; Sigma) and penicillin/streptomycin (50 units/mL and 50 µg/mL, respectively; Life Technologies). Rhesus macaque fibroblasts were cultured in DMEM supplemented with 10% FBS and penicillin/streptomycin (100 U/mL penicillin and 100 µg/mL streptomycin, respectively).

### Transient transfection

Transfections of HEK 293T cells were carried out in six-well plates, with cells at 50%–70% confluency, using GeneJuice (Merck) with 1 µg of total plasmid DNA per well, as per the manufacturer’s instructions.

### Gene inactivation by CRISPR/Cas9

Guide RNAs for TAP1, TAP2, and HM13 [Table S2 (27)] were inserted into pspgRNA [(28), Addgene plasmid #47108, a gift from Charles Gersbach]. HEK 293T cells were co-transfected with equal amounts of the pspgRNA plasmids and pCas9_GFP [(29), Addgene plasmid #44719, a gift from Kiran Musunuru]. EGFP-positive single cells were sorted 48 hours post transfection, and genomic DNA was isolated using QuickExtract (Lucigen). The gRNA binding sites were amplified using KOD Hot Start Polymerase (Merck), and the primers are shown in Table S3, with the following cycling conditions: 1 minute at 96°C; 5 cycles of 25 seconds at 96°C, 45 seconds at 70°C, and 45 seconds at 72°C; 21 cycles of 25 seconds at 96°C, 50 seconds at 65°C, and 45 seconds at 72°C; and 4 cycles of 25 seconds at 96°C, 60 seconds at 55°C, and 120 seconds at 72°C. PCR products were resolved on a 1.5% agarose gel and visualized with SYBRSafe (Life Technologies), purified using QIAquick spin columns (QIAGEN) and sequenced directly with both amplification primers. The mutations introduced by Cas9 were deconvoluted manually or using TIDE [https://tide.deskgen.com/ (30)]. Deconvolutions were confirmed by cloning the PCR products using a ZERO BLUNT PCR Cloning Kit (Life Technologies) and sequencing individual clones.

### Protein extraction and western blotting

Cell lysates were prepared by incubating cells on ice for 20 minutes in RIPA buffer (150 mM NaCl, 5 mM EDTA, 50 mM TRIS-Cl pH 8.0, 1% IGEPAL CA-630, 0.5% Sodium Deoxycholate, and 0.1% SDS) supplemented with cOmplete EDTA-free Protease Inhibitor Cocktail (Roche), followed by centrifugation at 13,000 rpm (4°C) for 10 minutes to remove organelle debris. Equal volumes of lysate were mixed with 2× LDS Loading Buffer (Life Technologies) and run on 10% Bis-Tris NuPage gels (Life Technologies) in 1× MOPS running buffer and then transferred to polyvinylidene difluoride (PVDF) membrane using 2× NOVEX Transfer Buffer (Life Technologies) and a Trans-Blot SD semi-dry transfer cell (Bio-Rad). Membrane blocking and antibody incubations were done at room temperature with constant mixing in 5% non-fat milk prepared from powder in PBS/0.1% Tween-20. Membranes were washed three times (5 minutes each) after each antibody incubation with PBS/0.1% Tween-20. Rabbit anti-SPP or mouse anti-Hepatitis C virus Core protein were detected using IRDye 800CW Donkey anti-Rabbit IgG or Goat anti-Mouse IgG (LiCor). The control antibody was DyLight 680 β-Tubulin Loading Control Monoclonal Antibody (Life Technologies). Membranes were visualized using a LICOR Odyssey X.

### HM13 reverse transcription PCR

One microgram of total cellular RNA prepared using RNeasy mini columns (QIAGEN) was reverse transcribed using EvoScript Universal cDNA Master (Roche). PCR amplification using the HM13 forward and reverse primers shown in Table S1 and electrophoresis, purification and cloning of the PCR products were all as before.

### Infection of rhesus macaque fibroblasts

The RhCMV strains used were as previously described: RhCMV 68-1-gag (31), RhCMV 68-1 ΔRh178 ΔRh182-189 [which lacks the known MHC class I evasion (18)], and RhCMV 68-1 ΔRh178 ΔRh182-189 ΔRh67 [which also lacks Rh67 (20)]. Rhesus macaque fibroblasts that expressed Mamu-A*01 were seeded in six-well plates and 5 × 10^5^ cells per well infected the following day in 500 µL serum-free media at a multiplicity of infection of 5 to ensure 100% infection. The cells were incubated at 37°C for 2 hours, then supplemented with complete medium, and cultured for a further 24 hours.

### Staining and flow cytometry

HEK 293T cells were stained 24 hours post transfection in 100 µL of Dulbecco’s modified PBS (DPBS, Sigma) at 4°C for 15 minutes with 3D12 (HLA-E) diluted 1:200, GAP.A3 (HLA-A*03:01) diluted 1:500, or W6/32 (pan-HLA) diluted 1:100, washed twice with DPBS, and fixed in 100 µL of Cytofix (BD Biosciences). Stained cells were acquired using an Attune NXT flow cytometer (Thermo Fisher) and analyzed using FlowJo 10 (BD Biosciences). Infected rhesus macaque fibroblasts were stained for 1 hour at 4°C in DMEM supplemented with 2.5% FBS (D2.5) with anti-Mamu-A*01 antibody at 5 ng/µL final concentration, a mouse monoclonal antibody specific for the RhCMV Rh152/151 Fc gamma receptor at 5 ng/µL final concentration, and Live/Dead Aqua (Thermo Fisher) at 0.5 µL/test. The cells were washed three times in D2.5 and then stained for 45 minutes at 4°C in D2.5 with an Alexa Fluor 647-conjugated goat anti-mouse IgG2a secondary antibody (Thermo Fisher) at 5 ng/µL final concentration to detect the RhCMV-infected cells. Cells were washed three times as before and fixed with 4% formaldehyde in PBS for 10 minutes at room temperature and then washed three times in PBS. Stained cells were acquired on a LSRII cytometer (BD Biosciences) and analyzed using FlowJo 10.

### Immunofluorescence microscopy

Immunofluorescence staining was carried out at room temperature, with the samples protected from light at all times after the addition of the secondary antibodies. HeLa cells stably expressing either HLA-A*03:01 (HeLa.A3) or HLA-E*01:03 (HeLa.E) (32) were washed twice with DPBS and then fixed for 15 minutes with 4% formaldehyde diluted in DPBS. Samples were permeabilized and blocked by incubating for 1 hour in blocking buffer [DPBS containing 0.1% (vol/vol) saponin and 2% (vol/vol) bovine serum albumin, both from Merck], stained with anti-calnexin antibody diluted in blocking buffer for 1.5 hours, washed three times with blocking buffer, stained with secondary antibody diluted in blocking buffer for 2 hours, washed as before, washed twice with DPBS, and then mounted with DAPI Fluoromount-G (Cambridge Bioscience). Images were obtained using a Zeiss LSM880 inverted confocal laser scanning microscope with a Plan-Apochromat 63×/1.4 oil objective and then processed and analyzed using ImageJ.

### Data analysis

Data were analyzed using Prism 10 (GraphPad), and details of the statistical tests used are given in the figure legends.

## RESULTS

### An experimental system to dissect up-regulation of HLA-E by UL40 and Rh67

Endogenous cell surface expression of HLA-E is very low, so we adopted the strategy used previously to show that Rh67 could increase surface expression of HLA-E (19). 293T cells were transiently transfected with a plasmid expressing a SCD of HLA-E*01:01 and β2-microglobulin (Fig. S1A), and expression was detected using the HLA-E-specific monoclonal antibody 3D12 (gating strategy shown in Fig. S1B). Consistent with previous reports regarding the relative cell surface expression levels of the two HLA-E alleles (33), the HLA-E*01:01 SCD was expressed at lower levels at the cell surface than the HLA-E*01:03 SCD (Fig. S1C), confirming that covalently linking β2-microglobulin to HLA-E does not significantly alter the normal expression of HLA-E.

**Fig 1:**
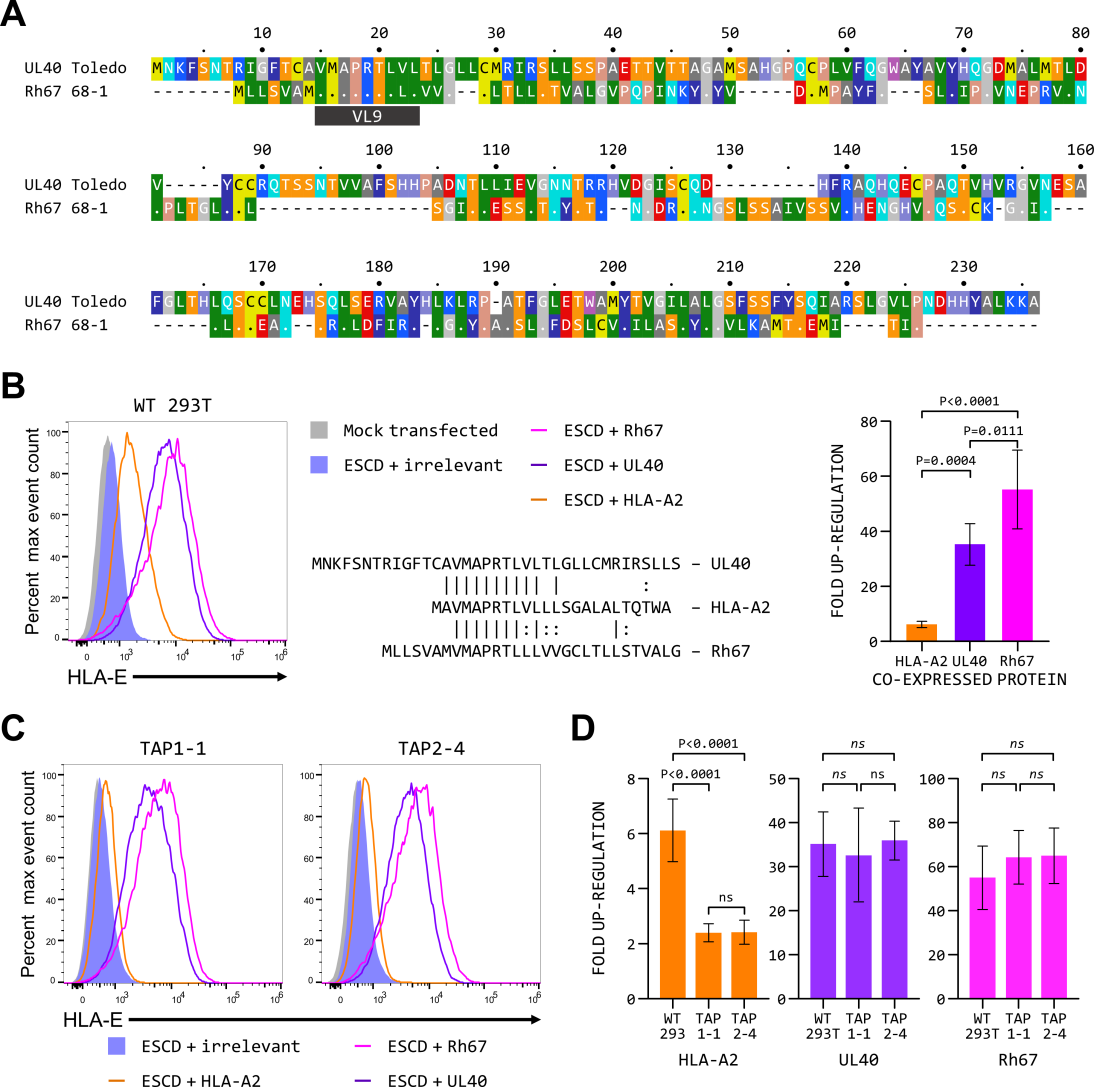
UL40 and Rh67 increase HLA-E expression by a TAP-independent pathway in 293T cells. (**A**) Alignment of the sequences of the HCMV UL40 gene (strain Toledo) and the Rhesus CMV Rh67 gene (strain 68-1). The location of the VL9 peptides, the only region of significant sequence similarity shared by the two proteins, is indicated. Amino acids are colored according to the standard scheme used by RasMol. (**B**) Representative HLA-E expression (of five independent replicates) in 293T cells co-transfected with the HLA-E*01:01 SCD expression plasmid and plasmids expressing an irrelevant protein (HLA-B*57:01; blue histogram), HLA-A*02:01 (orange line), UL40 (purple line), or Rh67 (pink line). The sequence alignment shows a comparison of the signal sequences of the transfected UL40, Rh67, and HLA-A*02:01 proteins. The graph shows the mean fluorescent intensity (±SD) for the five independent replicates. Statistical analysis was by one-way analysis of variance (ANOVA) with Tukey’s correction for multiple comparisons. (**C**) Representative HLA-E expression (of five independent replicates) in the TAP1-1 or TAP2-4 CRISPR/Cas9 knockout cell lines co-transfected with the HLA-E*01:01 SCD expression plasmid and plasmids expressing an irrelevant protein (HLA-B*57:01; blue histogram), HLA-A*02:01 (orange line), UL40 (purple line), or Rh67 (pink line). (**D**) Comparison of the fold up-regulation of HLA-E expression observed in the parental 293T cells and the TAP1 and TAP2 knockout cell lines when the HLA-E*01:01 SCD is co-expressed with HLA-A*02:01, UL40, or Rh67, relative to that of cells co-transfected with the HLA-E*01:01 SCD and the irrelevant protein, after subtraction of background staining of mock-transfected cells. Statistical analysis was performed using a one-way ANOVA with Tukey’s correction for multiple comparisons;. *ns* denotes not significant in panels **B and D**. Untransfected cells are shown as the gray-filled histograms in panels **B and C**.

Co-transfection of plasmids expressing either UL40 or Rh67 (protein sequences shown in Fig. 1A) with the HLA-E*01:01 SCD increases cell surface expression of HLA-E (Fig. 1B). In this system, up-regulation of HLA-E by Rh67 is always greater than that mediated by UL40 (~55-fold compared with ~35-fold; *P* = 0.0111), a difference that may simply reflect the fact that the Rh67 signal sequence appears to direct translation to the ER better than that of UL40 (Fig. S2A and D), which is presumably the essential first step in directing the VL9 peptide to the ER. Over-expression of HLA-A*02:01 results in a more modest increase in HLA-E expression (approximately sixfold). As the HLA-A*02:01 and UL40 signal sequences appear to be equivalent in directing translation to the ER (Fig. S2A and D), this lower up-regulation most likely results from the HLA-A*02:01 VL9 peptide being processed less efficiently than the VL9 peptides of UL40 and Rh67 in the cells used. Up-regulation of cell surface expression of the HLA-E*01:03 SCD is also seen with all three sources of the VL9 peptide (Fig. S1D), although the higher basal expression of HLA-E*01:03 means the fold increases are smaller than seen with HLA-E*01:01. Moreover, there is no difference in the magnitude of up-regulation seen with UL40 and Rh67 with HLA-E*01:03, most likely because of the increased stability of HLA-E*01:03 at the cell surface.

The UL40-mediated up-regulation of HLA-E expression does not require TAP (15, 16), and it has recently been shown that this is also true for the increase in MHC-E expression induced by Rh67 (20). To confirm that both UL40 and Rh67 were up-regulating HLA-E expression by a TAP-independent pathway in our 293T cells, we used CRISPR/Cas9 to inactivate the *TAP1* or *TAP2* genes (see Fig. S3; Tables S4 and S5 for details). For each, three single cell clones were established that had reduced MHC Ia expression consistent with the inactivation of TAP (Fig. S3C and D). Of these, clones TAP1-1 and TAP2-4 (genetic lesions shown in Fig. S3E and F) were confirmed TAP deficient (Fig. S3G through I). For both the TAP1-1 and TAP2-4 clones, we observed a significant reduction in the up-regulation of HLA-E expression mediated by co-expression of HLA-A*02:01, but not UL40 and Rh67 (Fig. 1C and D). This confirms that delivery of the Rh67 peptide to HLA-E does not require transit from the cytosol to the ER lumen via TAP in 293T cells, allowing these cells to be used to dissect the sequences required for the TAP-independent increase in HLA-E expression mediated by Rh67.

### The N-terminal portion of the Rh67 signal sequence is critical for TAP-independent up-regulation of HLA-E

Up-regulation of HLA-E by UL40 is known to only require the UL40 signal sequence, with the 13 amino acids between the start codon and the UL40 VL9 peptide being both necessary for TAP-independent up-regulation of HLA-E by the UL40 VL9 peptide and sufficient to confer TAP independence on the VL9 peptide present in the HLA-A*02:01 signal sequence (16). We confirmed that the UL40 sequence behaves identically in our 293T cells (Fig. S4) and then undertook investigation of the sequence architecture of the Rh67 signal sequence.

**Fig 2:**
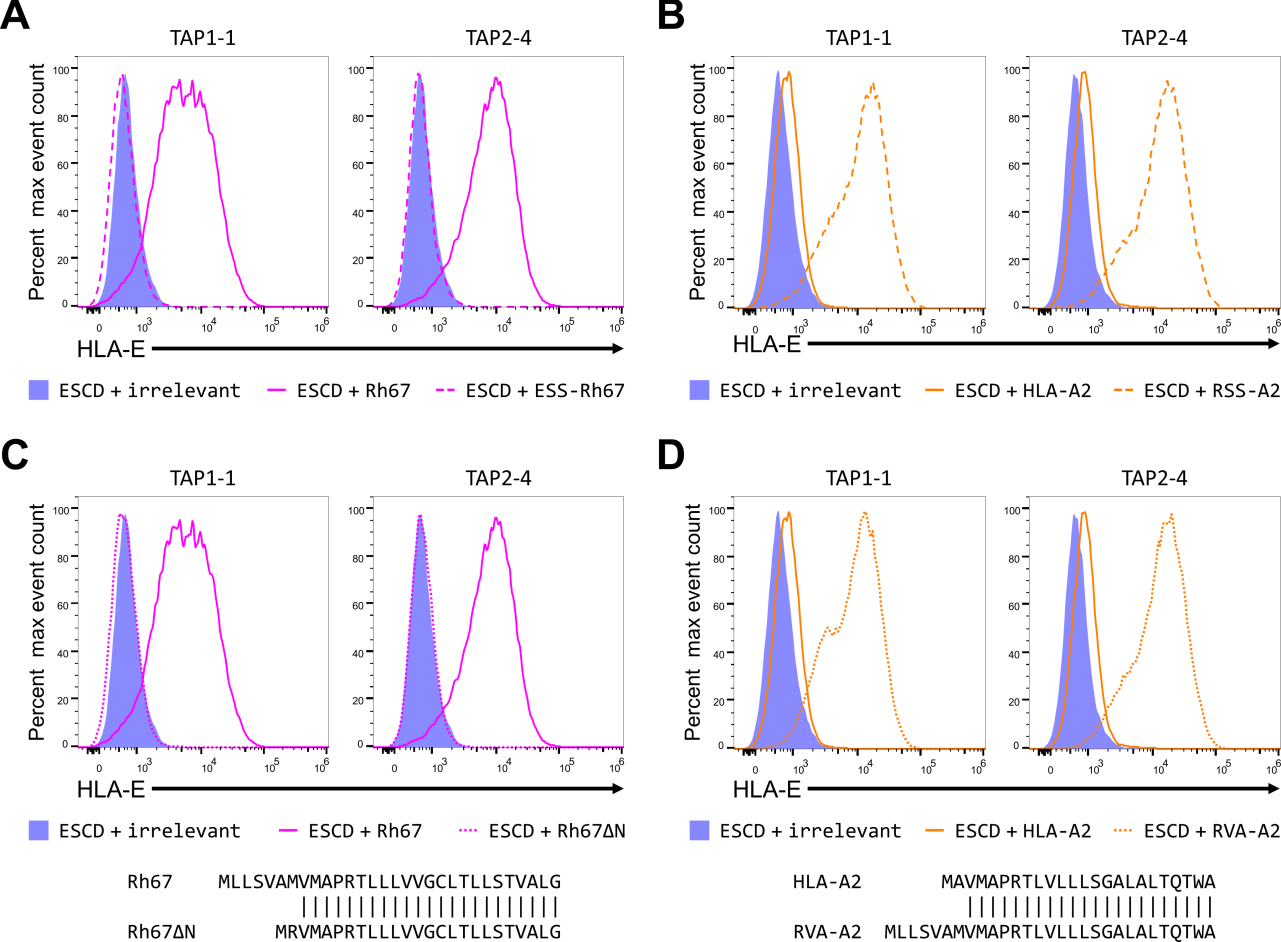
The N terminus of the Rh67 signal sequence is critical for TAP-independent up-regulation of HLA-E. (**A**) Representative HLA-E expression (of five independent replicates) in clone TAP1-1 or clone TAP2-4 co-transfected with the HLA-E*01:01 SCD expression plasmid and plasmids expressing an irrelevant protein (HLA-B*57:01, blue-filled histogram), Rh67 (pink line), or Rh67 with the HLA-E signal sequence (ESS-Rh67, broken pink line). (**B**) Representative HLA-E expression (of five independent replicates) in clone TAP1-1 or clone TAP2-4 co-transfected with the HLA-E*01:01 SCD expression plasmid and plasmids expressing an irrelevant protein (HLA-B*57:01, blue-filled histogram), HLA-A*02:01 (orange line), HLA-A*02:01 (orange line), or HLA-A*02:01 with the Rh67 signal sequence (RSS-A2, broken orange line). (**C**) Representative HLA-E expression (of five independent replicates) in clone TAP1-1 or clone TAP2-4 transfected with the HLA-E*01:01 SCD expression plasmid and plasmids expressing an irrelevant protein (HLA-B*57:01, blue-filled histogram), Rh67 (pink line), or Rh67 with the N-terminal truncation shown in the sequence alignment (Rh67ΔN, dotted pink line). (**D**) Representative HLA-E expression (of five independent replicates) in clone TAP1-1 or clone TAP2-4 transfected with the HLA-E*01:01 SCD expression plasmid and plasmids expressing an irrelevant protein (HLA-B*57:01, blue-filled histogram), HLA-A*02:01 (orange line), or HLA-A*02:01 with the first two amino acids of the signal sequence replaced with the first seven amino acids of the Rh67 signal sequence (RVA-A2, dotted orange line).

As expected, replacing the Rh67 signal sequence with that of HLA-E (which does not contain the VL9 peptide) abolished up-regulation of HLA-E (ESS-Rh67; Fig. 2A) in the TAP knockout cell lines, whereas replacing the mature Rh67 coding sequence with that of HLA-A*02 did not (RSS-A2; Fig. 2B). These results confirm that, as with UL40, the signal sequence of Rh67 is sufficient to mediate the up-regulation of HLA-E surface expression and that the mature protein is not required.

The Rh67 signal sequence has only has six amino acids between its start codon and its VL9 peptide, and this sequence is less polar than the equivalent 13 amino acids in UL40. Despite this, replacing the six intervening amino acids in the Rh67 signal sequence with a single arginine (to preserve the expected signal sequence charge distribution, as done previously with UL40) abolished up-regulation of HLA-E by Rh67 in both the *TAP1* and *TAP2* knockout cells (Rh67ΔN; Fig. 2C). Although this change has a modest effect on the functioning of the Rh67 signal sequence (Fig. S2B and D), this is not sufficient to explain the loss of HLA-E up-regulation. Conversely, replacing the single two amino acids (MA) upstream of the HLA-A*02:01 VL9 peptide with the first seven amino acids from the N terminus of the Rh67 signal sequence again had little effect on the functioning of the HLA-A*02:01 signal sequence (Fig. S2C and D) but allowed TAP-independent up-regulation of HLA-E expression by the HLA-A*02:01 VL9 peptide (RVA-A2; Fig. 2D). Thus, despite differing in both sequence composition and length from the corresponding sequence in UL40, the N terminus of the Rh67 signal sequence is both necessary for the TAP independence of the Rh67 VL9 peptide and sufficient to confer TAP-independence on the HLA-A*02:01 VL9 peptide.

### Signal peptide peptidase is required for the processing of the UL40 and Rh67 VL9 peptides

Processing of the VL9 peptide from the MHC Ia signal sequences requires cleavage of the signal sequence downstream of the VL9 peptide by SPP (3). Although it was initially reported that the UL40 signal sequence was not a substrate for SPP (34), a later report suggested that it could be processed by SPP or an SPP-like protease (16), albeit inefficiently. However, it is not clear if such cleavage is required to generate the UL40 VL9 peptide, and the UL40 signal sequence may have evolved to be a poor substrate if cleavage by SPP prevents correct processing of the UL40 VL9 peptide. Therefore, to address whether SPP is required for processing of the VL9 peptides in the UL40 and Rh67 signal sequences, we used CRISPR/Cas9 to knock out *HM13*, the gene that encodes SPP.

The *HM13* gRNA used targets the end of exon 4 (with the Protospacer Adjacent Motif overlapping the 5′ splice site), which encodes the longest cytoplasmic loop of SPP (Fig. S5A). Four single-cell clones were selected that had no SPP protein detectable by western blot (Fig. S5B). Three of these clones had lesions in the *HM13* gene that would be predicted to have significant effects on expression of functional protein (Fig. S5C; Table S6). The exception (clone HM13-2) had a 10-bp deletion in one allele (resulting in premature termination of translation in exon 5) and the in-frame deletion of the final 9 bp of exon 4 in the other. Although this clone could still potentially express SPP with a three-amino acid deletion, the altered sequence at the end of the exon is predicted to have a significant effect on the recognition of the adjacent 5′ splice site (Table S7), potentially affecting protein expression as a result of inefficient or aberrant splicing.

**Fig 3:**
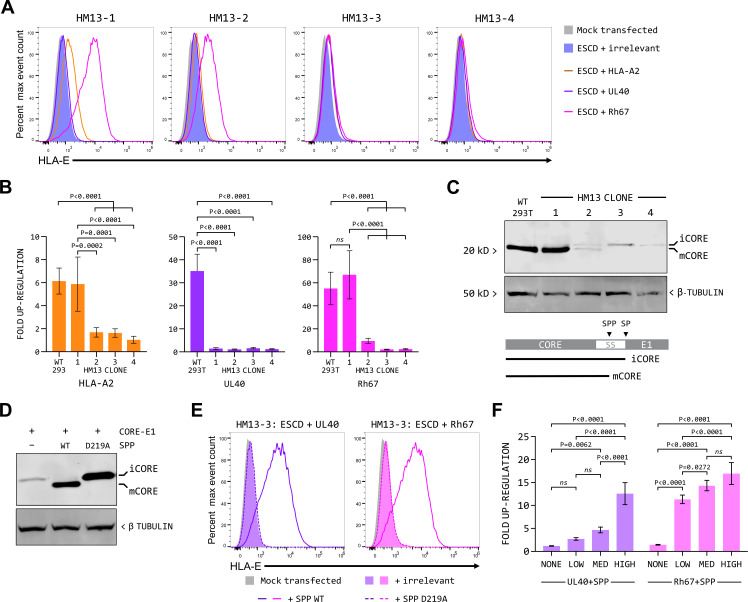
Cleavage by SPP is essential for the up-regulation of HLA-E mediated by UL40 and Rh67. (**A**) Representative HLA-E expression (of five independent replicates) in the four HM13 knockout CRISPR/Cas9 single-cell clones co-transfected with the HLA-E*01:01 SCD expression plasmid and plasmids expressing an irrelevant protein (HLA-B*57:01, blue-filled histogram), HLA-A*02:01 (orange line), UL40 (purple line), or Rh67 (pink line). (**B**) Comparison of HLA-A*02:01, UL40, and Rh67 mediated up-regulation of expression of the HLA-E*01:01 SCD in the parental cells (see Fig. 1) or the HM13 CRISPR/Cas9 clones. Statistical analysis of fold up-regulation, calculated as shown in Fig. 1, for the five independent replicates was by one-way ANOVA with Tukey’s correction for multiple comparisons; *ns* denotes not significant. (**C**) Western blot analysis using an antibody against the HCV core protein of lysates from the parental (WT) 293T cells and the four HM13 CRISPR/Cas9 single-cell clones expressing the HCV Core-E1 precursor protein. The schematic shows the arrangement of the CORE and E1 proteins, which are separated by a signal sequence (SS), and the cleavage sites of signal peptidase and SPP. The bands corresponding to the immature (iCORE) core-E1 precursor and the mature (mCORE) core proteins are indicated. (**D**) Western blot analysis using an antibody against the HCV core protein of lysates from clone HM13-3 transfected with the core-E1 expression plasmid and plasmids expressing an irrelevant protein (HLA-B*57:01, lane 1), wild-type SPP (lane 2), or the catalytically inactive SPP mutant (D219A, lane 3). (**E**) Representative HLA-E expression (of five independent replicates) in clone HM13-3 cells transfected with plasmids expressing the HLA-E*01:01 SCD and UL40 (left-hand graph, colored purple) or the HLA-E*01:01 SCD and Rh67 (right-hand graph, colored pink), as well as plasmids expressing an irrelevant protein (HLA-B*57:01, filled colored histograms), wild-type SPP protein (solid colored lines), or the catalytically inactive D219A SPP mutant (dashed colored lines). (**F**) Relative up-regulation of HLA-E expression (of five independent replicates) of HM13-3 cells transfected with the HLA-E*01:01 SCD, UL40, or Rh67 and three different amounts of the SPP expression plasmid were used (93 ng, 187 ng, and 375 ng for “low,” “med,” and “high,” respectively). Statistical analysis of five independent replicates was by one-way ANOVA with Tukey’s correction for multiple comparisons. In addition to the statistical significance indicated on the graph, HLA-E up-regulation was significantly higher for Rh67 than UL40 for each amount of SPP plasmid used (*P* values < 0.0001, <0.0001, and 0.0003 for low, medium, and high, respectively); *ns* denotes not significant. Mock-transfected cells are shown as the gray-filled histograms in panels A and D.

Despite the lack of detectable SPP protein in all four clones, we observed differences in their ability to support up-regulation of surface expression of the HLA-E*01:01 (Fig. 3A and B) and HLA-E*01:03 (Fig. S5D and E). While none of the clones supported up-regulation by UL40, two clones (HM13-1 and HM13-2) still supported up-regulation by Rh67. For HM13-1, the level of up-regulation induced by Rh67 was indistinguishable from that in the parental cells for both HLA-E*01:01 and HLA-E*01:03. For clone HM13-2, we consistently observed lower levels of up-regulation by Rh67, although this was only statistically significantly higher than the up-regulation observed with clones HM13-3 and HM13-4 for HLA-E*01:03. The more modest up-regulation of HLA-E expression by the HLA-A*02:01 VL9 peptide, which has previously been shown to be dependent on cleavage by SPP, was similarly affected. Up-regulation in clone HM13-1 was indistinguishable from that in the parental cells and significantly reduced in the other three clones for both HLA-E*01:01 and HLA-E*01:03.

Clones HM13-3 and HM13-4 were also unable to support maturation of the HCV Core-E1 precursor protein [which is cleaved by SPP (35)], with only the immature iCore protein being detected (Fig. 3C). In contrast, clone HM13-1 was indistinguishable from the parental cells, with only the mature Core (mCore) protein observed. Clone HM13-2 had an intermediate phenotype, with both mCore and iCore being detected. We conclude that clones HM13-1 and HM13-2 still retain some level of SPP activity, while clones HM13-3 and HM13-4 are truly SPP deficient. Consistent with this, processing of the HCV Core-E1 precursor could be restored in clone HM13-3 by transiently over-expressing wild-type SPP (Fig. 3D, lane 2). In contrast, expression of a catalytically inactive mutant (in which the aspartic acid residue at position 219 was mutated to alanine, D219A) stabilized the iCore protein but did not support its processing to mCore (Fig. 3D, lane 3).

The failure of UL40 and Rh67 to up-regulate expression of HLA-E in clones HM13-3 and HM13-4 suggests that processing of both the UL40 and Rh67 VL9 peptides is dependent on SPP. As with processing of the HCV Core-E1 precursor, up-regulation of HLA-E by both UL40 and Rh67 could be rescued in clone HM13-3 by transiently over-expressing wild-type SPP but not the D219A mutant (Fig. 3E). Consistent with the UL40 signal sequence being a poor substrate for SPP, we observed that the up-regulation of HLA-E by UL40 was more sensitive than that of Rh67 to the amount of SPP expression plasmid used in these transfections (Fig. 3F). These results confirm that cleavage by SPP is essential for generating the VL9 peptides from both the UL40 and Rh67 signal sequences.

We hypothesized that up-regulation of HLA-E expression by Rh67 but not UL40 in clones HM13-1 and HM13-2 most likely resulted from these cells retaining low levels of SPP activity, either insufficient to support processing of the UL40 VL9 peptide or with substrate specificity altered by the CRISPR/Cas9 lesions. It proved possible to amplify SPP cDNA from clone HM13-1 (Fig. S6A), and several novel isoforms were identified by sequencing (Table S8). The predominant isoform (HM13-1 cDNA D) was almost identical to SPP isoform 1 except for the sequence of exon 4 (which encodes the longest cytoplasmic loop) mostly being replaced by a cryptic exon from within intron 3 (Fig. S6B). Over-expression of this isoform in clone HM13-3 rescued up-regulation of HLA-E by both UL40 and Rh67 (Fig. S6C), confirming that this novel isoform could recognize both signal sequences. Therefore, the impairment to UL40-mediated up-regulation in clone HM13-1 most likely stems from the low levels of expression of the novel SPP isoform (below the limit of detection for western blotting with the SPP antibody used) being sufficient to allow processing of the VL9 peptide of Rh67, but not that of the UL40 VL9 peptide. A similar situation likely persists in clone HM13-2, where a very low level of SPP expression may occur from the HM13 allele with the in-frame deletion adjacent to the exon 4 splice site.

### Improving cleavage of the UL40 signal sequence by SPP increases up-regulation of HLA-E

**Fig 4:**
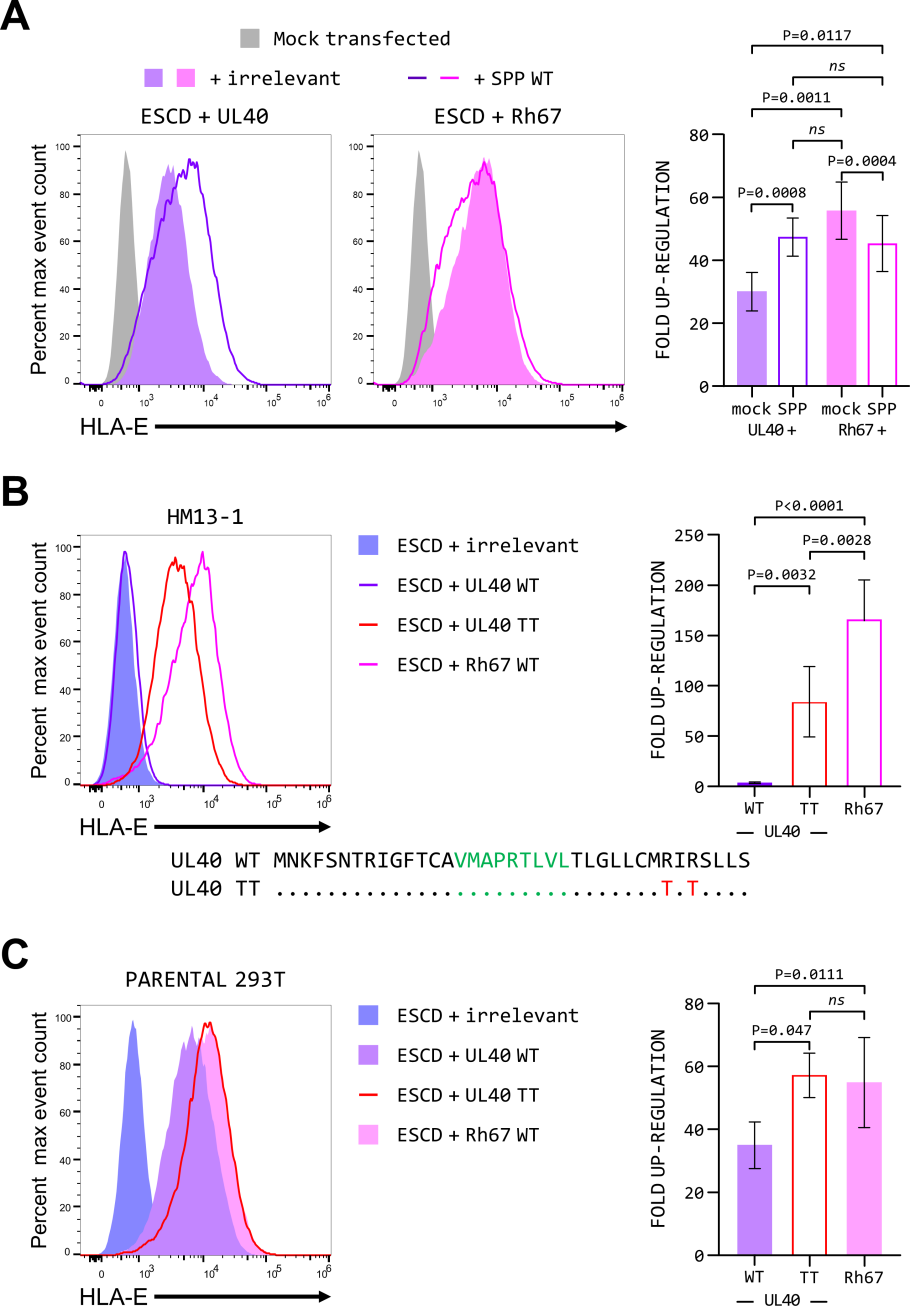
Inefficient processing of the UL40 signal sequence by SPP affects up-regulation of HLA-E. (**A**) Representative HLA-E expression (of five independent replicates) in 293T cells transfected with the HLA-E*01:01 SCD expression plasmid and plasmids expressing UL40 (left-hand graph, purple) or Rh67 (right-hand graph, pink), as well as plasmids expressing an irrelevant protein (HLA-B*57:01 filled histogram) or wild-type SPP protein (colored line). Mock-transfected cells are shown as the gray-filled histograms. (**B**) Representative HLA-E expression in clone HM13-1 (of five independent replicates) transfected with the HLA-E*01:01 SCD expression plasmid and plasmids expressing an irrelevant protein (HLA-B*57:01, blue-filled histogram), wild-type UL40 (purple line), the UL40 signal sequence mutant (TT, red line), or wild-type Rh67 (pink line). (**C**) Up-regulation of HLA-E expression of five independent replicates of the parental 293T cells transfected with the HLA-E*01:01 SCD expression plasmid and plasmids expressing an irrelevant protein (HLA-B*57:01, blue-filled histogram), wild-type UL40 (purple-filled histogram), the UL40 TT mutant (red line), or wild-type Rh67 (pink-filled histogram). Statistical analysis of fold up-regulation (calculated as for Fig. 1) was by one-way ANOVA with Tukey’s correction for multiple comparisons; *ns* denotes not significant.

Consistent with the UL40 signal sequence being a poor substrate for SPP, over-expression of wild-type SPP in the parental 293T cells resulted a significant increase in up-regulation of HLA-E by UL40 (Fig. 4A, left histogram). For Rh67, over-expression of SPP had the opposite effect, with a slight but reproducible decrease in HLA-E expression (Fig. 4A, right histogram), perhaps resulting from over-processing of the Rh67 signal sequence reducing the amount of VL9 peptide generated.

These results suggested that the increase of surface expression by UL40 may be limited by inefficient SPP cleavage. To confirm this, we tested the effects of a previously described mutant of the UL40 signal sequence in which the arginines at positions 31 and 33 are mutated to threonines (34), which was shown to convert the UL40 signal sequence into an efficient SPP substrate. Despite having no effect on the functioning of the UL40 signal sequence (Fig. S7), we observed up-regulation of HLA-E when this mutant form of UL40 was co-expressed with the HLA-E*01:01 SCD in clone HM13-1 (Fig. 4B), as well as a significant increase in the up-regulation of HLA-E by the mutant UL40 signal sequence compared with the wild-type signal sequence in the parental 293T cells (Fig. 4C). These data suggest that the UL40 signal sequence has evolved to be a sub-optimal substrate for SPP compared with the signal sequence of Rh67 and that this has the effect of limiting the increase in HLA-E expression mediated by the UL40 VL9 peptide.

### The mature UL40 and Rh67 proteins reduce surface expression of MHC class I proteins

**Fig 5:**
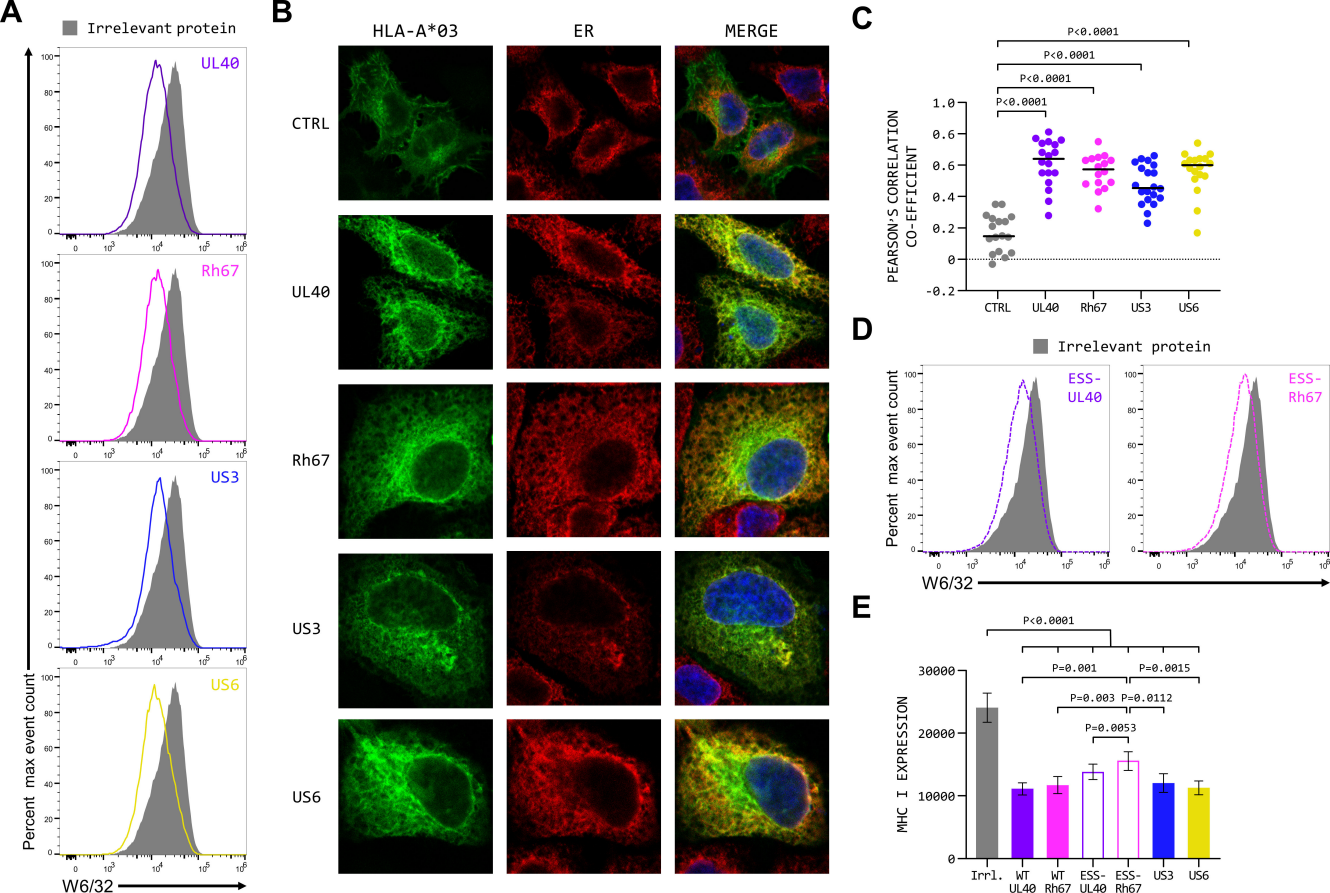
The mature UL40 and Rh67 proteins decrease surface expression of classical MHC class I proteins. (**A**) Representative MHC class I protein expression (W6/32 staining) of five independent replicates of 293T cells transfected with plasmids expressing an irrelevant protein (KIR2DS2; dark-gray histogram), UL40 (purple line), Rh67 (pink line), US3 (blue line), or US6 (yellow line). (**B**) Representative micrographs of HeLa cells stably expressing HLA-A*03:01 (HeLa.A3) either untransfected (CTRL) or transiently transfected with plasmids expressing UL40, Rh67, US3, or US6. ER was visualized with an antibody specific for calnexin. (**C**) Quantification of colocalization of HLA-A*03:01 with ER in the transfected HeLa.A3 cells. Statistical analysis was by one-way ANOVA with Tukey’s correction for multiple comparisons. (**D**) Representative MHC class I protein expression (W6/32 staining) of five independent replicates of 293T cells transfected with plasmids expressing an irrelevant protein (KIR2DS2; dark-gray histogram), ESS-UL40 (dashed purple line), or ESS-Rh67 (dashed pink line). (**E**) Statistical analysis was by one-way ANOVA with Tukey’s correction for multiple comparisons. Only those comparisons that were statistically significant are shown.

No function has been ascribed to the mature UL40 and Rh67 proteins, and the lack of significant sequence homology beyond the VL9 peptide means that it is not clear if these proteins share a function. However, in the course of these studies, we observed that expression of UL40 or Rh67 resulted in a significant reduction in the cell surface MHC Ia expression on 293T cells, comparable to that induced by the HCMV US3 and US6 proteins (Fig. 5A and E). Expression of UL40, Rh67, US3, or US6 in HeLa cells stably expressing HLA-A*03:01 with a C-terminal EGFP tag [HeLa.A3 (32)] resulted in a significant increase in ER retention of the HLA-A*03:01 (Fig. 5B and C), suggesting the down-regulation results (at least in part) from an impediment to the MHC Ia protein exiting the ER. In contrast, only UL40 and Rh67 (and not US3 or US6) altered the ER association of HLA-E in HeLa cells stably expressing EGFP-tagged HLA-E*01:03 [HeLa.E (32); Fig. S8A and B].

Comparison of the levels of MHC class I protein expressed by the parental 293T cells and the SPP-deficient clones HM13-3 and HM13-4 (Fig. S9A) indicated that the down-regulation of MHC class I expression induced by UL40 and Rh67 did not result from reduced SPP availability as a result of over-expression of UL40 or Rh67. Consistent with this, UL40 and Rh67 induced similar levels of MHC class I downregulation in clones HM13-3 and HM13-4 (Fig. S9B), which lack functional SPP. Finally, while the up-regulation of HLA-E by UL40 and Rh67 depends exclusively on the signal sequences of these proteins, a significant decrease in surface expression of MHC class I protein was also observed when the signal sequence swapped versions of UL40 and Rh67 were expressed (ESS-UL40 and RSS-UL40; Fig. 5D and E), suggesting that this down-regulation is a function of the mature UL40 and Rh67 proteins rather than the signal sequence.

### Deletion of Rh67 alters MHC class I down-regulation in RhCMV-infected fibroblasts

**Fig 6:**
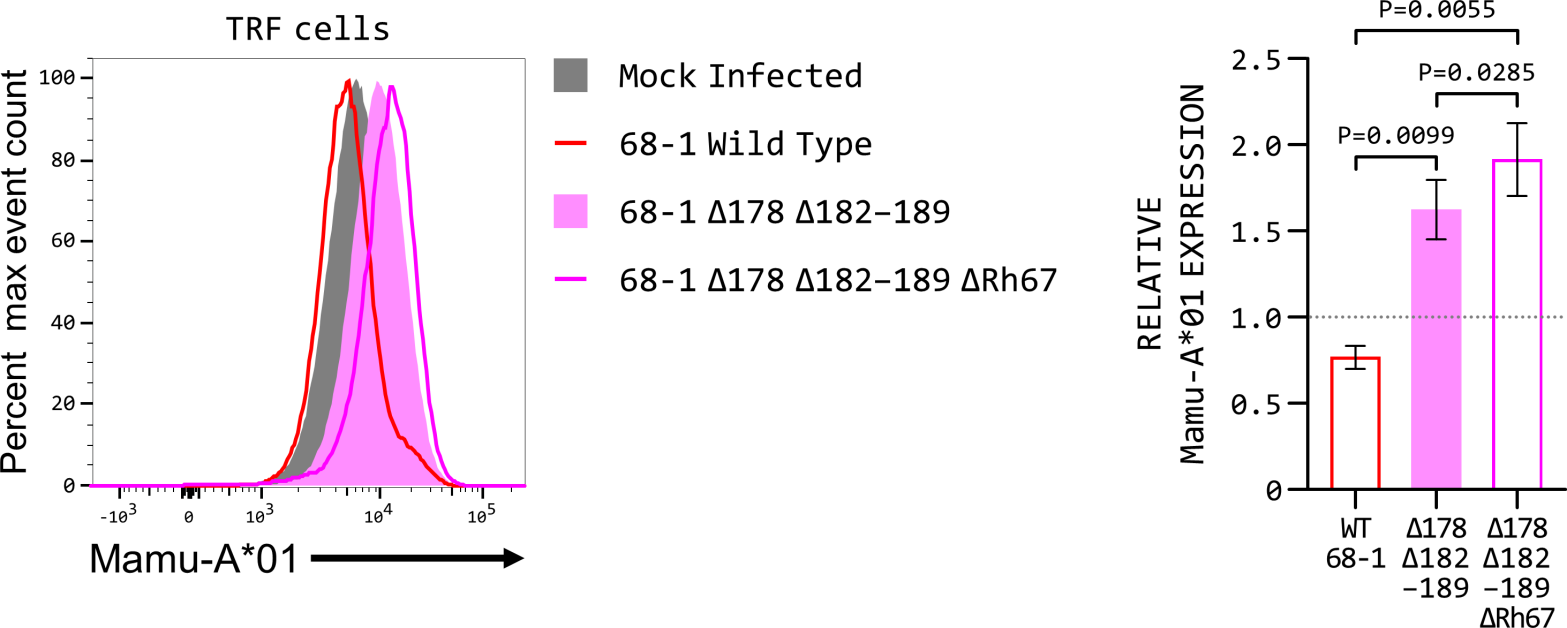
Deletion of Rh67 counteracts some of the MHC class I down-regulation in RhCMV-infected cells. Representative Mamu-A*01 expression (of four independent replicates) in macaque fibroblasts infected with three different RhCMV strains: 68-1 (red line), 68-1 with the Rh178 and Rh182–189 genes deleted (pink-filled histogram), and 68-1 with Rh67 deleted in addition to Rh178 and Rh182–189 (pink line). Mock-infected cells are shown as the gray-filled histogram. The graph shows quantitation of Mamu-A*01 expression by the infected cells, relative to that of the mock-infected cells. Statistical analysis was by repeated measures ANOVA with Tukey’s correction for multiple comparisons.

Analysis of MHC class I expression in primary Rhesus macaque fibroblasts infected with different mutants of RhCMV confirmed that this down-regulation was not an artefact of transfected cells and is also evident in RhCMV-infected *ex vivo* cultures (Fig. 6). Fibroblasts infected with RhCMV 68-1 showed reduced surface expression of Mamu-A*01 compared with mock-infected cells. In contrast, Mamu-A*01 expression was higher in cells infected with RhCMV 68-1 lacking the known MHC class I evasion genes (ΔRh178 and ΔRh182–Rh189) and higher again in cells infected with virus that also had the Rh67 gene deleted. These results confirm that the mature Rh67 protein represents a previously undescribed component of the RhCMV immune evasion arsenal, contributing to the down-regulation of MHC class I expression at the surface of infected cells.

### The mature UL40 and Rh67 proteins also reduce surface expression of HLA-E

**Fig 7:**
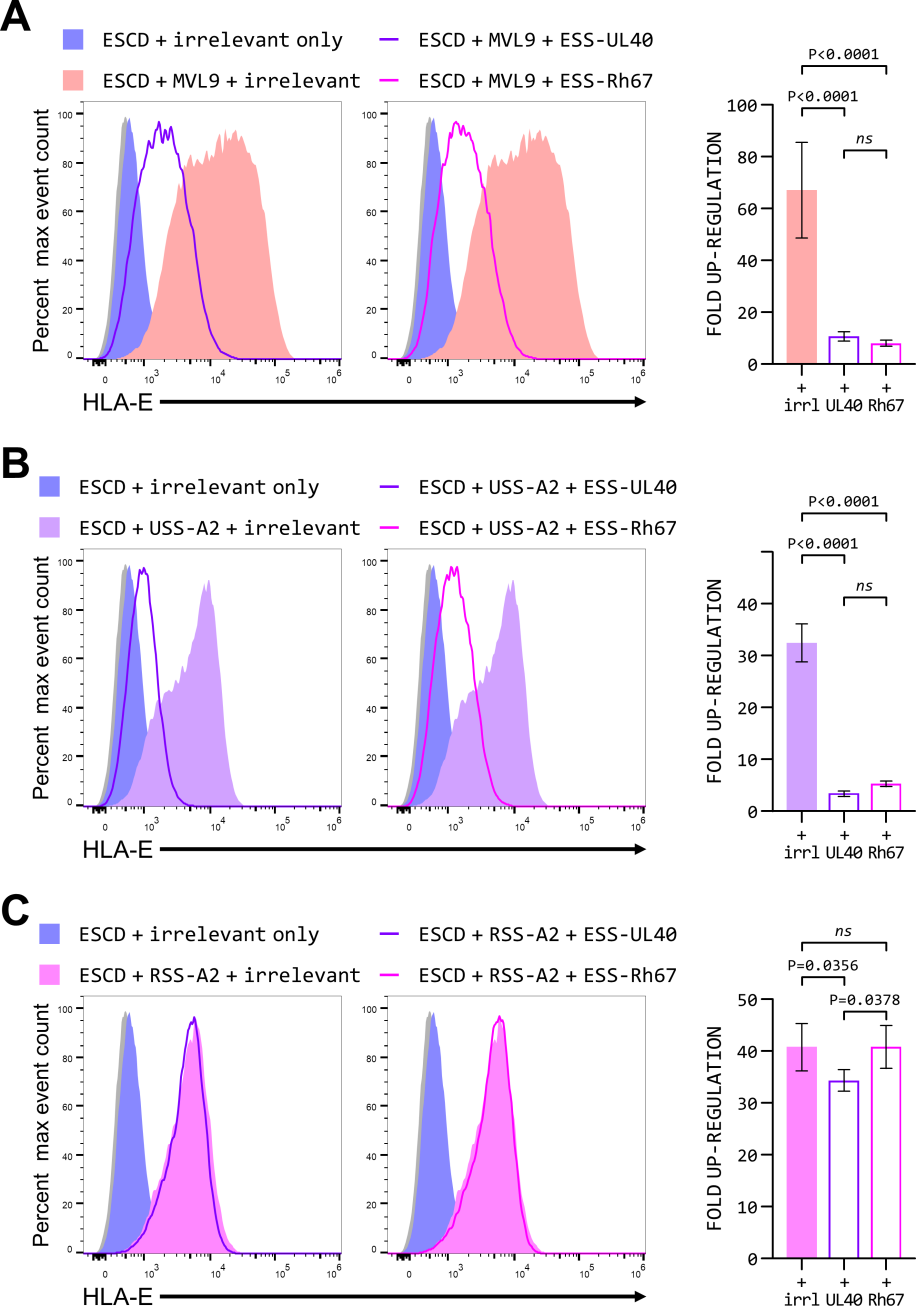
The mature UL40 and Rh67 proteins can counteract some but not all HLA-E up-regulation. (**A**) Representative HLA-E expression (of five independent replicates) in 293T cells co-transfected with the HLA-E*01:01 SCD expression plasmid and the following combinations of plasmids: a plasmid expressing only an irrelevant protein (KIR2DS2, blue-filled histogram), plasmids expressing VL9 as a 10mer peptide with an initiating methionine (MVL9) and the irrelevant protein (red-filled histogram), plasmids expressing UL40 with the HLA-E signal sequence (ESS-UL40) and MVL9 (purple line), or plasmids expressing Rh67 with the HLA-E signal sequence (ESS-Rh67) and MVL9 (pink line). (**B**) Representative HLA-E expression (of five independent replicates) in 293T cells co-transfected with the HLA-E*01:01 SCD expression plasmid and the following combinations of plasmids: a plasmid expressing only an irrelevant protein (KIR2DS2, blue-filled histogram), plasmids expressing HLA-A*02:01 with the UL40 signal sequence (USS-A2) and the irrelevant protein (purple-filled histogram), plasmids expressing UL40 with the HLA-E signal sequence (ESS-UL40) and USS-A2 (purple line), or plasmids expressing Rh67 with the HLA-E signal sequence (ESS-Rh67) and USS-A2 (pink line). (**C**) Representative HLA-E expression (of five independent replicates) in 293T cells co-transfected with the HLA-E*01:01 SCD expression plasmid and the following combinations of plasmids: a plasmid expressing only an irrelevant protein (KIR2DS2, blue-filled histogram), plasmids expressing HLA-A*02:01 with the Rh67 signal sequence (RSS-A2) and the irrelevant protein (pink-filled histogram), plasmids expressing UL40 with the HLA-E signal sequence (ESS-UL40) and RSS-A2 (purple line), or plasmids expressing Rh67 with the HLA-E signal sequence (ESS-Rh67) and RSS-A2 (pink line). In all panels, mock-transfected cells are shown as the gray-filled histograms, and the total amount of plasmid DNA was kept constant in each transfection. Statistical analysis in each case was by one-way ANOVA with Tukey’s correction for multiple comparisons; *ns* denotes not significant.

The reduction in MHC class I surface expression observed when UL40 or Rh67 was expressed was not limited to classical MHC class I. The level of HLA-E expressed at the cell surface when the HLA-E*01:01 SCD was co-expressed with the VL9 peptide (as a 10mer in the cytosol with an initiating methionine—see Fig. S3G for details) was significantly reduced when either ESS-UL40 or ESS-Rh67 were also expressed (Fig. 7A). Although HLA-E expression was higher in the presence of ESS-UL40 or ESS-Rh67 than when the HLA-E*01:01 SCD was co-expressed with an irrelevant protein, down-regulation of HLA-E expression by the mature UL40 and Rh67 proteins was unexpected given the up-regulation induced by their signal sequence.

We therefore investigated whether the mature UL40 and Rh67 proteins also affected the up-regulation of HLA-E resulting from their own signal sequences. Surface expression of the HLA-E*01:01 SCD in the presence of the UL40 signal sequence (linked to HLA-A*02:01, USS-A2) was reduced by co-expression of either ESS-UL40 or ESS-Rh67 (Fig. 7B), although HLA-E expression was again higher than when the HLA-E*01:01 SCD was expressed alone. This suggests that the level of expression of HLA-E in HCMV-infected cells will represent a balance between the competing processes mediated by the UL40 signal sequence and the mature UL40 protein. In contrast, the up-regulation of HLA-E expression caused by the Rh67 signal sequence (when RSS-A2 was co-expressed) was only modestly affected by expression of the mature UL40 or Rh67 proteins (Fig. 7C). At present, it is not clear what underpins this difference in the sensitivity of the UL40 and Rh67 VL9 peptides to inhibition by the mature UL40 and Rh67 proteins. It may suggest that expression of Mamu-E on the surface of RhCMV-infected cells may be higher than that of HLA-E on the surface of HCMV-infected cells.

## DISCUSSION

We previously showed that Rh67 was likely to be the RhCMV functional homolog of the HCMV UL40 protein (19), a conclusion confirmed by demonstration that the up-regulation of MHC-E expression mediated by Rh67 does not require TAP (20). We have now extended our characterization of Rh67 to show striking functional similarity with UL40, despite the sequences of the two proteins showing little sequence homology. In particular, the architecture of the signal sequences appears identical: both possess N-terminal sequences that are essential for the up-regulation of HLA-E and that confer TAP-independence on the HLA-A*02:01 VL9 peptide when placed at the start of the HLA-A*02 signal sequence. Moreover, despite the extensive differences in the flanking sequences, processing of both the UL40 and Rh67 VL9 peptides also depends on cleavage by the host protease SPP.

Perhaps most surprising, however, our results also suggest that the mature UL40 and Rh67 proteins contribute to the broader immune evasion orchestrated by CMV by decreasing the surface expression of classical MHC class I proteins. For both UL40 and Rh67, the reductions in cell surface MHC class Ia protein levels were also accompanied by an increase in the co-localization of MHC class Ia protein with the ER. This suggests that the mechanism of down-regulation (at least in part) depends on limiting exit of the protein from the ER. For Rh67, we have confirmed that this decrease in surface expression also occurs during infection with RhCMV: deletion of the Rh67 gene resulted in a significant increase in surface MHC class I expressed on cells infected with a RhCMV recombinant lacking the known MHC-I down-regulating proteins. For UL40, our results must be considered more preliminary, and it will be important to confirm that the effects of UL40 on MHC class I expression are also observed in cells infected with HCMV.

Our results also showed that, in certain situations, the mature UL40 and Rh67 proteins can both reduce the surface expression of HLA-E. The down-regulation observed when the VL9 peptide was expressed in the cytosol (Fig. 7A) may stem from the more general disruption of MHC class I expression that the mature proteins also appear to mediate. For UL40, however, the effect on HLA-E also appears to counteract the increase in HLA-E expression mediated by its own signal sequence VL9 peptide, although we still observed a net increase in HLA-E expression. In contrast, the Rh67 VL9 peptide was unaffected by the action of the mature Rh67 or UL40 proteins. As with the effect on global MHC class I expression, it will be critical to determine if the antagonism of the UL40 VL9 peptide by the mature UL40 protein also manifests in cells infected with HCMV.

The UL40 signal sequence is processed less efficiently by SPP than the Rh67 signal sequence, and this may also reduce the magnitude of HLA-E up-regulation mediated by UL40. This difference could simply reflect the fact that the UL40 signal sequence appears less able to direct nascent translation to the ER membrane (Fig. S2), thereby resulting in a reduced amount of UL40 signal sequence available for SPP to process. However, the mutation that improves the UL40 signal sequence as a substrate for SPP (Fig. 4) has little effect on its functioning as a signal sequence (Fig. S7), suggesting that the reduced processing by SPP does result directly from the UL40 signal sequence being a poor SPP substrate.

One explanation for these differences is that UL40 may have evolved to limit the expression of HLA-E on the surface of infected cells. Perhaps related to this, the most potent peptide ligand for NKG2/CD94 interaction (VMAPRTLFL, from the HLA-G signal sequence) is rarely found in the UL40 signal sequence (36), in contrast to the other MHC Ia VL9 variants (such as VMPARTLLL, VMAPRTLIL, VMAPRTVL, and VMAPRTVLL). Limiting HLA-E expression may be necessary to prevent recognition by NK cells expressing the activating NKG2C/CD94 ligand, which has a lower affinity for HLA-E than the inhibitory NKG2A/CD94 complex (37). The fact that Rh67 does not appear to limit its own VL9 suggests that Mamu-E expression by RhCMV-infected cells may be higher than HLA-E expression by HCMV-infected cells, perhaps reflecting differences in immune surveillance between human and macaque NK cells and the expression levels required to trigger CD94/NKG2 signaling. Perhaps consistent with this idea is the recent finding that duplication of the Mamu-E locus appears to be common in rhesus macaques (38).

At present, it is not clear how the UL40 and Rh67 VL9 peptides reach the lumen of the ER in the absence of TAP. The TAP dependence of the MHC class I VL9 peptides results from SPP cleaving the signal sequence downstream of the VL9 peptide, releasing the VL9-containing fragment back into the cytosol (3). The simplest mechanism for the TAP independence of the UL40 and Rh67 VL9 peptides, therefore, would be for SPP to cleave upstream of VL9, releasing the precursor directly into the lumen of the ER. Prediction of SPP cleavage sites is complicated by the lack of a consensus sequence, although it is likely that helix breaking or polar residues following a stretch of alpha helix are critical (39). While the N terminus of the Rh67 signal sequence is sufficiently hydrophobic to tolerate being embedded in the ER membrane, as required for cleavage by SPP, that of UL40 is not (containing more polar residues). It was previously proposed that the N terminus of the UL40 signal sequence would initially project into the cytosol and undergo a topological change to allow cleavage by SPP (16). No direct evidence in support of this model was presented, and it requires the UL40 signal sequence adopting a type I orientation, while SPP typically cleaves proteins in the type II orientation (40).

Sequences similar to that cleaved by SPP in the HLA-A*02 signal sequence (LLSG, immediately following the VL9 peptide) lie both upstream (LLSV) and downstream (LLCT) of the Rh67 VL9 peptide. The first of these is present immediately after the start codon and thus may not be suitably positioned for cleavage by SPP. For UL40, the only match in the signal sequence (LLCM) lies downstream of the VL9 peptide. Therefore, for both signal sequences, it seems likely that SPP cleaves downstream of the VL9 peptide, which would release the VL9 precursors back into the cytosol, thereby requiring subsequent transit across the ER membrane. The first five amino acids of the Rh67 signal sequence are identical to those of a known TAP-independent epitope (MLLVSPLLL), which corresponds to the first 10 amino acids of the calreticulin signal sequence (41). This signal sequence can be cleaved by SPP (34), which would release a fragment containing this epitope into the cytosol. It has been proposed that this peptide can enter the ER in the absence of TAP by virtue of its extremely hydrophobic sequence (16). The fragment generated by SPP cleaving after the Rh67 VL9 peptide would also be extremely hydrophobic (perhaps MLLSVAMVMAPRTLLLVV) and may behave in the same way as the calreticulin peptide. In contrast, the longer peptide that would be generated by cleavage of the UL40 signal sequence after the VL9 peptide would be significantly less hydrophobic and presumably enters the lumen of the ER by a different mechanism, perhaps explaining the sensitivity of the UL40 VL9 peptide to the inhibitory effect of the mature UL40 and Rh67 proteins.

Having shown a requirement for SPP cleavage, we are also interested in determining what other host factors are required to process the mature VL9 peptide from both UL40 and Rh67. If precursors of these peptides do return to the cytosol, they may require processing at the C terminus by the proteasome, as has been observed for the VL9 peptides from classical MHC class I alleles (5). Removal of the amino acids upstream of the Rh67 and UL40 VL9 peptides is also presumably required, which may suggest the involvement of the ER-resident aminopeptidases ERAP1 or ERAP2. Formation of the MHC Ia VL9 peptide also requires removal of two amino acids from the N terminus of the signal sequence, but the enzyme responsible has yet to be identified. Disruption of ERAAP (the homolog of ERAP1) in mice has been shown to alter the peptide presented by Qa-1, the murine homolog of HLA-E (42). Rather than presenting the Qdm epitope (AMAPRTLLL) from the murine class leader sequence, ERAAP-deficient cells present a peptide derived from the Fam49b protein (FYAEATPML). However, it is not clear if this results from the absence of ERAAP preventing correct processing of the Qdm peptide or the Fam49b peptide no longer being destroyed. Two different ERAP1 inhibitors have been shown to have no effect on surface expression of HLA-E (5), suggesting that ERAP1 may not be responsible for trimming the class I VL9 peptide in humans. A role for ERAP1 may be unlikely for the UL40 VL9 peptide however, as microRNAs encoded in the HCMV US4 (43) and UL112 (44) genes have been suggested to downregulate expression of some (but not all) isoforms of ERAP1. No equivalent microRNAs have been described for RhCMV.

The sequence conservation between the mature UL40 and Rh67 proteins is similar to that of the other HCMV/RhCMV immunomodulatory proteins [US2/Rh182, US3/Rh184, US6/Rh185, US8/Rh186, US10/Rh187, and US11/Rh189, which show 21%–30% sequence identity and 33%–43% sequence similarity (17)]. Both proteins are known to localize in the ER (20), but it is not clear if they are associated with the membrane or present in the lumen. The TMHMM webserver (45) predicts a single transmembrane domain for Rh67, but not for UL40, and homology scanning using InterProScan (46) fails to identify any known functional domains. The structures of these proteins predicted by AlphaFold3 (47) also differ (Fig. S10), although both are predicted to have an alpha helical C terminal projection, which in the case of UL40 is followed by a disordered region. Importantly, the overall confidence of these predictions is low. For UL40, the per atom confidence estimates (pLDDT) only reach “confident” for the globular core of the structure, with the rest of the protein only classed as “low” or “very low.” This is reflected in the overall predicted template modeling score (pTM) of 0.51, which is only just above the threshold (0.5) to suggest the prediction is similar to the actual structure of the protein. For Rh67, the prediction is even less confident, with an overall pTM score of just 0.24 and the majority of the structure having “low” or “very low” pLDDT scores. Determining if these proteins are indeed functional homologs will require identification of the step(s) of the antigen processing and presentation pathway that they target.

Evasion of NK cells by the maintenance of MHC-E expression during CMV infection appears to be common throughout primate evolution, and VL9 peptides are found in all but one of the known primate CMV genomes (Table S9). As with UL40 and Rh67, the viral proteins that contain the peptide show very little sequence homology (Fig. S11). In each case, the peptide is predicted to be present in a signal sequence, and its position reflects that of Rh67, with only the HCMV UL40 protein having an extended N-terminus. It seems likely that these primate CMV proteins will also mediate TAP-independent up-regulation of MHC-E expression, and the associated mature proteins may also play a role in decreasing surface expression of MHC class I proteins. We note that all but one of these primate CMV VL9 peptides share the same sequence (VMAPRTLLL), including a variety of CMV specific from both Old World and New World monkeys. With the exception of macaques, chimpanzees, and baboons, it is currently impossible to assess how diverse the MHC Ia VL9 sequences are in most of the species presented in Table S9 as only very small numbers of alleles have been sequenced. For Rhesus macaques, the latest release of the non-human primate MHC database (3–12 January 2024) shows that the MHC Ia VL9 is highly conserved, with only a small number of Mamu-B alleles deviating from leucine at positions 7–9 (Fig. S12). A similar situation is also observed with baboon MHC Ia VL9 peptides (Fig. S13), although the number of alleles sequenced is substantially smaller than for rhesus macaques. This contrasts with the polymorphic nature of the VL9 peptide in HCMV UL40, which reflects the diversity of HLA VL9 peptides, and suggests that conservation of the CMV VL9 sequences may reflect the lack of host VL9 variation. However, sequencing of additional primate CMV isolates (and MHC class I alleles) will be required to determine if the VL9 peptides present in the UL40 homologs are indeed invariant, as well as to confirm that this conservation is indeed a consequence of a similar lack of polymorphism in the VL9 peptides present in the MHC Ia alleles.

The one known exception to the invariance of the VL9 peptide sequence present in the sequenced primate CMVs is seen with chimpanzee CMV, where the peptide sequence (TMAPKTLLI, TL9) differs from that present in all the other primate CMVs at three positions. Of these, the lysine at position 5 is the most striking difference as interaction of HLA-E with CD94/NKG2A is abrogated by mutating position 5 of the VL9 peptide from arginine to lysine (48). Chimpanzees are the closest living relatives of humans, and their CD94 and NKG2A genes share >96% sequence identity with their human counterparts (49). Coupled with the fact that all sequenced Patr-A, B, and C alleles have VL9 peptides with arginine at position 5 (Fig. S14), it would be expected that monitoring of class I expression by NK cells via the MHC-E/CD94/NKG2 axis will be identical in humans and chimpanzees. This similarity with humans also extends to the diversity of C termini that are present in the MHC I VL9 peptides. As would be expected, the chimpanzee CMV UL40 protein is also the most closely related to HCMV UL40 of all the primate orthologs, although the sequence upstream of the TL9 peptide in chimpanzee CMV is shorter than that of HCMV, and not as hydrophobic as that in the other NHP CMVs. To date, only two annotated chimpanzee CMV sequences have been published (50, 51), and both are of the same isolate. Given that this strain was cultured *in vitro* for several years prior to sequencing (50), it remains to be determined if this sequence is indeed representative of chimpanzee CMV UL40 and if it truly reflects differences in how chimpanzee CMV evades detection by NK cells.

In conclusion, we show that despite the similarities in how the signal sequences of UL40 and Rh67 up-regulate HLA-E expression (including the location of the critical sequences and processing of their VL9 peptides being dependent on cleavage by SPP), there are also important differences between these two proteins. Processing of the UL40 VL9 peptide is less efficient than that of Rh67, and the resulting increase in HLA-E expression appears to be partially counteracted by the ability of the mature UL40 protein to inhibit surface expression of MHC class I. The mature Rh67 protein is also able to inhibit MHC class I expression but does not affect the up-regulation of HLA-E mediated by its own signal sequence VL9 peptide. Such differences may have important implications for the ability of a HCMV-vectored vaccine to induce protective HLA-E-restricted responses (analogous to the protective Mamu-E responses elicited by the RhCMV 68.1 SIV vaccine), and further investigation into the similarities and differences of how HCMV and RhCMV manipulate both classical and non-classical MHC expression is warranted.

## Data Availability

All of the data relating to this study are included in the article and supplemental material.

## SUPPLEMENTAL MATERIAL

Supplemental material jvi.01206-24-s0001.pdf Fig. S1 to S14; Tables S1 to S9; supplementary references.

ASM does not own the copyrights to Supplemental Material that may be linked to, or accessed through, an article. The authors have granted ASM a non-exclusive, world-wide license to publish the Supplemental Material files. Please contact the corresponding author directly for reuse.
