# Supplementary material for: Regulation of the cell surface expression of classical and non-classical MHC proteins by the human cytomegalovirus UL40 and rhesus cytomegalovirus Rh67 proteins
Source: J Virol. 2024 Aug 29;98(9):e01206-24. doi: 10.1128/jvi.01206-24 (PMC11406984; doi:10.1128/jvi.01206-24)
Supplement: Supplemental material — Fig. S1 to S14; Tables S1 to S9; supplementary references. [file jvi.01206-24-s0001.pdf]

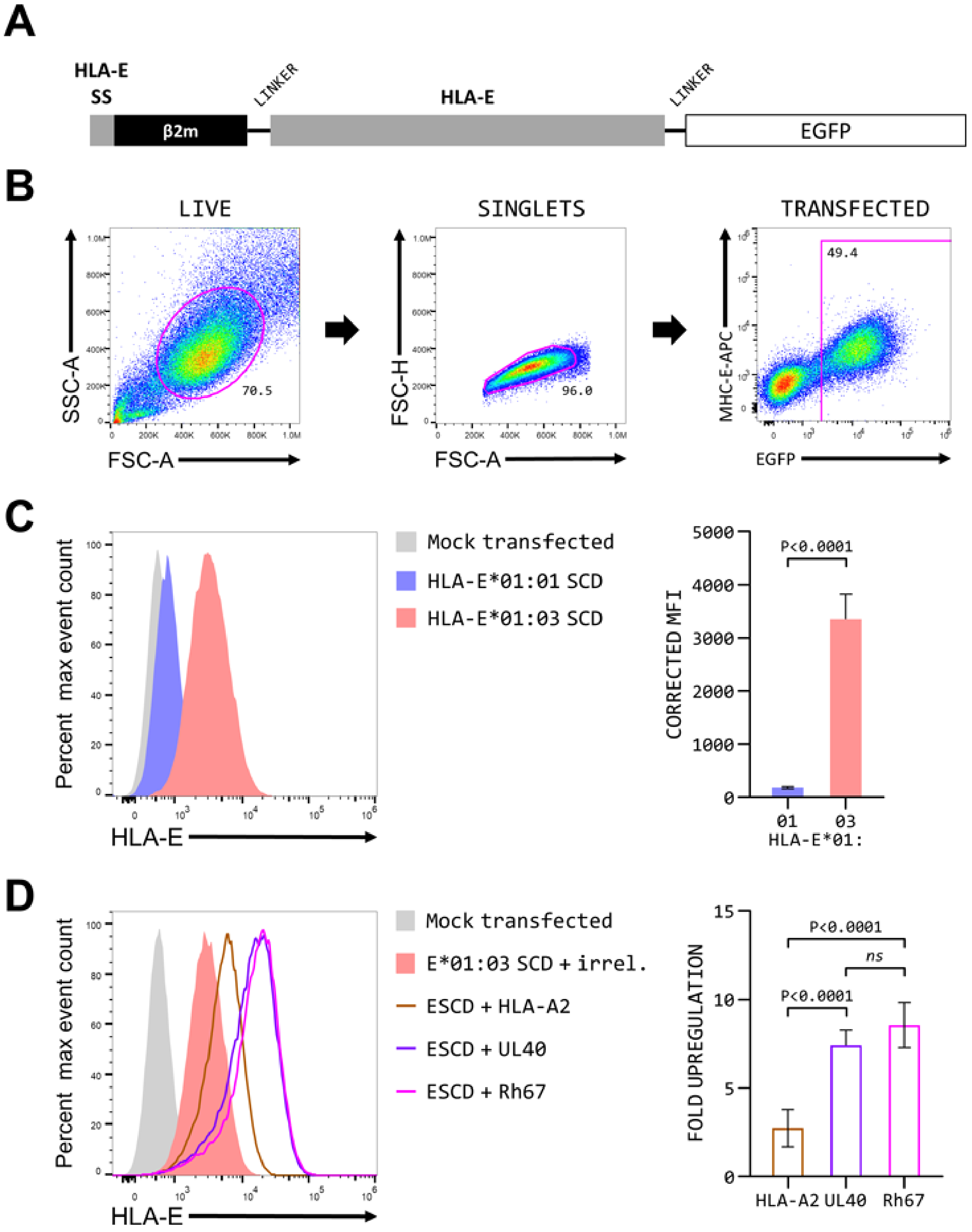

SUPPLEMENTARY FIGURE 1: Assaying up-regulation of HLA-E expressed as single chain dimers with β2-microglobulin

SUPPLEMENTARY FIGURE 1: Assaying up-regulation of HLA-E expressed as single chain dimers with β2-microglobulin

(A) Schematic of the HLA-E single chain dimer (SCD) design, which comprises the HLA-E signal sequence (SS), the mature coding sequence of β2-microglobulin (β2m), a flexible Glycine-Serine linker ($[GGGGS]_4$), the mature coding sequence of HLA-E*01:01 (arginine at position 107) or HLA-E*01:03 (glycine at position 107), a second short linker (DPVAT), and EGFP. **(B)** Gating of transfected 293T cells on the Attune NXT: debris was excluded by side scatter area (SSC-A) against forward scatter area (FSC-A), singlets defined by forward scatter height (FSC-H) against FSC-A, and transfected cells by expression of the EGFP tag. The example shown is gating of cells transfected with a plasmid expressing the HLA-E*01:03 SCD. **(C)** Representative HLA-E expression (of five independent replicates) in 293T cells co-transfected with the HLA-E*01:01 and HLA-E*01:03 SCD (filled blue and red histograms, respectively). The graph shows the mean fluorescent intensity (MFI) ±SD for the five independent replicates after subtraction of the MFI of the mock transfected cells (filled grey histogram). **(D)** Representative HLA-E expression (of five independent replicates) in 293T cells co-transfected with the HLA-E*01:03 SCD expression plasmid and plasmids expressing an irrelevant protein (HLA-B*57:01; filled red histogram), UL40 (purple line), or Rh67 (pink line). Fold upregulation for UL40 and Rh67 was calculated relative to expression in the presence of the irrelevant control protein. In both panels **C** and **D**, statistical significance was assessed by unpaired two-tailed T tests; *ns* denotes not significant.

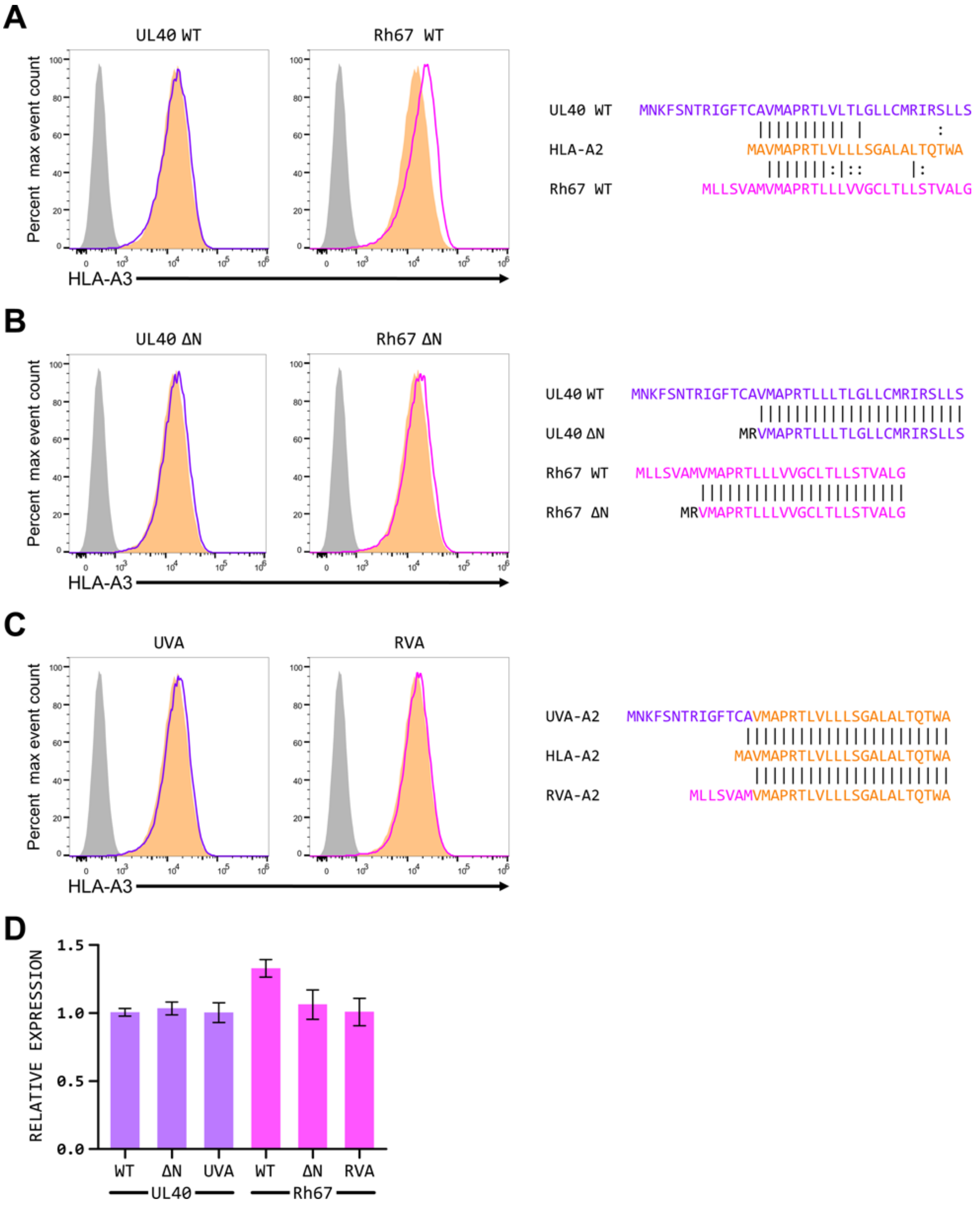

SUPPLEMENTARY FIGURE 2: Comparison of the HLA-A*02:01, UL40, Rh67, and mutant/hybrid signal sequences

SUPPLEMENTARY FIGURE 2: Comparison of the HLA-A*02:01, UL40, Rh67, and mutant/hybrid signal sequences

The various wild type and mutant or hybrid signal sequences used in this work were confirmed functional by their ability to direct expression of a single chain dimer (SCD) of HLA-A*03:01 to the cell surface (assessed by staining using the GAP.A3 antibody). HLA-A*03:01 was used as (unlike HLA-A*02:01) it cannot present the VL9 peptide, so surface expression of HLA-A*03:01 will not be affected by the presence or absence of the VL9 peptide in a signal sequence (nor by the efficiency with which the VL9 peptide is generated). In panels **A–C**, expression of the HLA-A*03:01 SCD with the HLA-A*02:01 signal sequence is shown by filled orange histograms, and expression of the HLA-A*03:01 SCD with UL40 and Rh67-derived signal sequences shown as the purple and pink lines as follows: **(A)** the wild type UL40 and Rh67 signal sequences; **(B)** the UL40 and Rh67 signal sequences with the N terminus deleted (ΔN), as used in Supplementary Figure 4 and Figure 3; and **(C)** the HLA-A*02:01 signal sequence with the transplanted UL40 and Rh67 N-terminal sequences (UVA and RVA, respectively), as used in Supplementary Figure 4 and Figure 3. **(D)** Quantitation of the surface expression of the HLA-A*03:01 SCD with each of the tested signal sequences, relative to that obtained with the HLA-A*02:01 signal sequence, for the 5 independent replicates. Only expression with the Rh67 signal sequence differed significantly from that observed with all the other signal sequences tested ($P \leq 0.0001$ in each case; one way ANOVA with Tukey's correction for multiple comparisons).

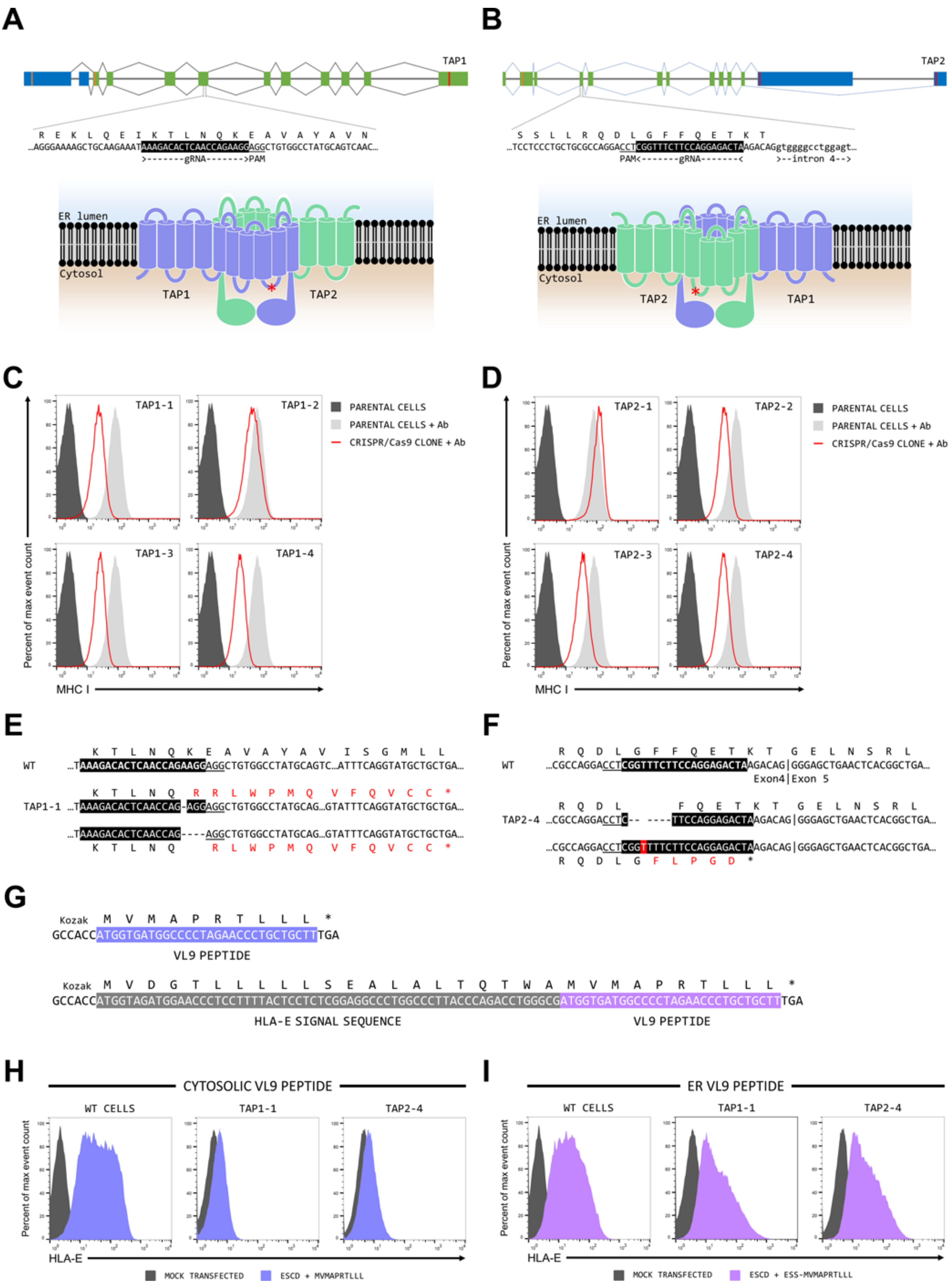

SUPPLEMENTARY FIGURE 3: Inactivation of TAP1 or TAP2 by CRISPR/Cas9

SUPPLEMENTARY FIGURE 3: Inactivation of TAP1 or TAP2 by CRISPR/Cas9

(A, B) Schematics of the TAP1 and TAP2 genes and cartoons of the TAP complex, showing the sequences (white on black, with the adjacent Protospacer Adjacent Motif [PAM] underlined) and locations (red asterisks on the cartoons) of the CRISPR/Cas9 gRNAs target sequences. **(C**, **D)** W6/32 staining of the single cell clones obtained using the TAP1 and TAP2 guide RNAs. Unstained cells are shown as dark grey filled histograms, stained parental cells as light grey filled histograms, and the stained single cell clones as the red lines. **(E**, **F)** Details of the genetic lesions in the two alleles of TAP1 in clone TAP1-1, or in the two alleles of TAP2 in clone TAP2-4, which were selected for use in this study. The locations of the gRNA target sequences are highlighted (white text on black), with the adjacent PAM sequences underlined. For TAP1-1, one allele has a single base deletion in the target sequence, the other a 4 base deletion starting that removes the last three bases of the target sequence and the first base of the PAM. Both lesions result in the use of the same cryptic stop codon (in the same exon as the target sequence) after the translation of 19 or 18 novel amino acids (respectively). For TAP2-4, one allele has a single base insertion in the target sequence, which results in the use of a downstream cryptic stop codon in the same exon after translation of 5 novel amino acids. The other allele has a 6 base deletion starting in the target sequence, that deletes 2 amino acids from the third cytoplasmic domain of the protein, which has been implicated in peptide binding (Supplementary Reference 1). Given the reduction in W6/32 staining of TAP2-4 is similar to that of the clones with complete disruption of both alleles, it would appear that this in-frame deletion is sufficient to abolish TAP function. **(G)** Sequences of the peptide minigene constructs used to express the VL9 peptide in either the cytosol (top), or the lumen of the ER using the HLA-E signal sequence (bottom). **(H)** Representative HLA-E expression in the parental 293T cells (left-hand graph), clone TAP1-1 (central graph) or clone TAP2-4 (right-hand graph) following transfection with the HLA-E*01:01 SCD expression plasmid and a plasmid expressing the VL9 peptide in the cytosol (filled blue histograms). **(I)** Representative HLA-E expression in the parental 293T cells (left-hand graph), clone TAP1-1 (central graph) or clone TAP2-4 (right-hand graph) following transfection with the HLA-E*01:01 SCD expression plasmid and a plasmid expressing the VL9 peptide with the HLA-E signal sequence (filled purple histograms). In panels **H** and **I**, mock transfected cells are shown as the dark grey filled histograms.

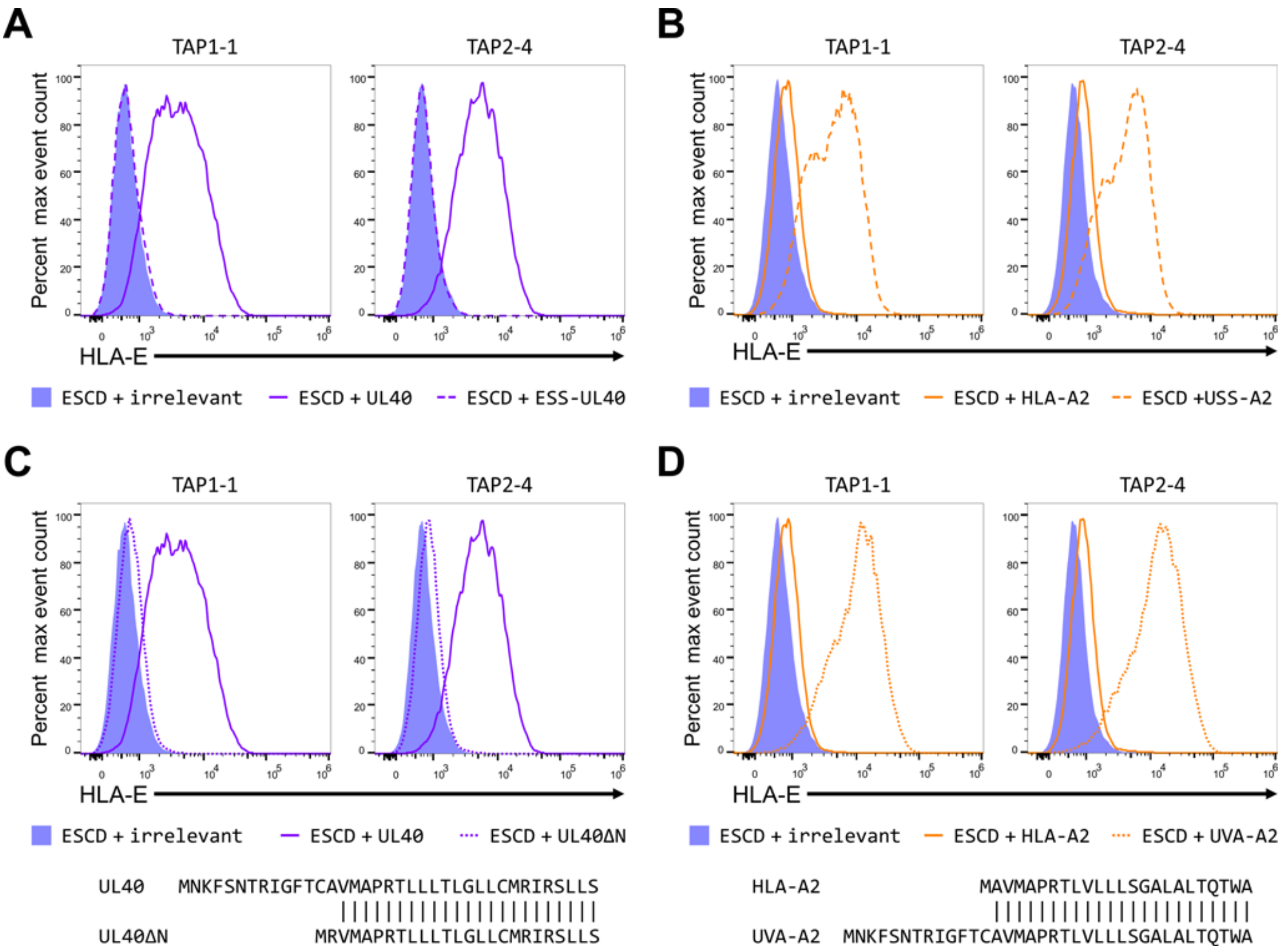

SUPPLEMENTARY FIGURE 4: The N terminus of the UL40 signal sequence is critical for TAP-independent up-regulation of HLA-E

(A) Representative HLA-E expression (of five independent replicates) in clone TAP1-1 or clone TAP2-4 co-transfected with the HLA-E*01:01 SCD expression plasmid and plasmids expressing an irrelevant protein (HLA-B*57:01, blue filled histogram), UL40 (purple line), or UL40 with the HLA-E signal sequence (ESS-UL40, broken purple line). **(B)** Representative HLA-E expression (of five independent replicates) in clone TAP1-1 or clone TAP2-4 co-transfected with the HLA-E*01:01 SCD expression plasmid and plasmids expressing an irrelevant protein (HLA-B*57:01, blue filled histogram), HLA-A*02:01 (orange line), HLA-A*02:01 (orange line), or HLA-A*02:01 with the UL40 signal sequence (USS-A2, broken orange line). **(C)** Representative HLA-E expression (of five independent replicates) in clone TAP1-1 or clone TAP2-4 transfected with the HLA-E*01:01 SCD expression plasmid and plasmids expressing an irrelevant protein (HLA-B*57:01, blue filled histogram), UL40 (purple line), or UL40 with the N-terminal truncation shown in the sequence alignment (UL40ΔN, dotted purple line). **(D)** Representative HLA-E expression (of five independent replicates) in clone TAP1-1 or clone TAP2-4 transfected with the HLA-E*01:01 SCD expression plasmid and plasmids expressing an irrelevant protein (HLA-B*57:01, blue filled histogram), HLA-A*02:01 (orange line), or HLA-A*02:01 with the first two amino acids of the signal sequence replaced with the first 14 amino acids of the UL40 signal sequence (UVA-A2, dotted orange line).

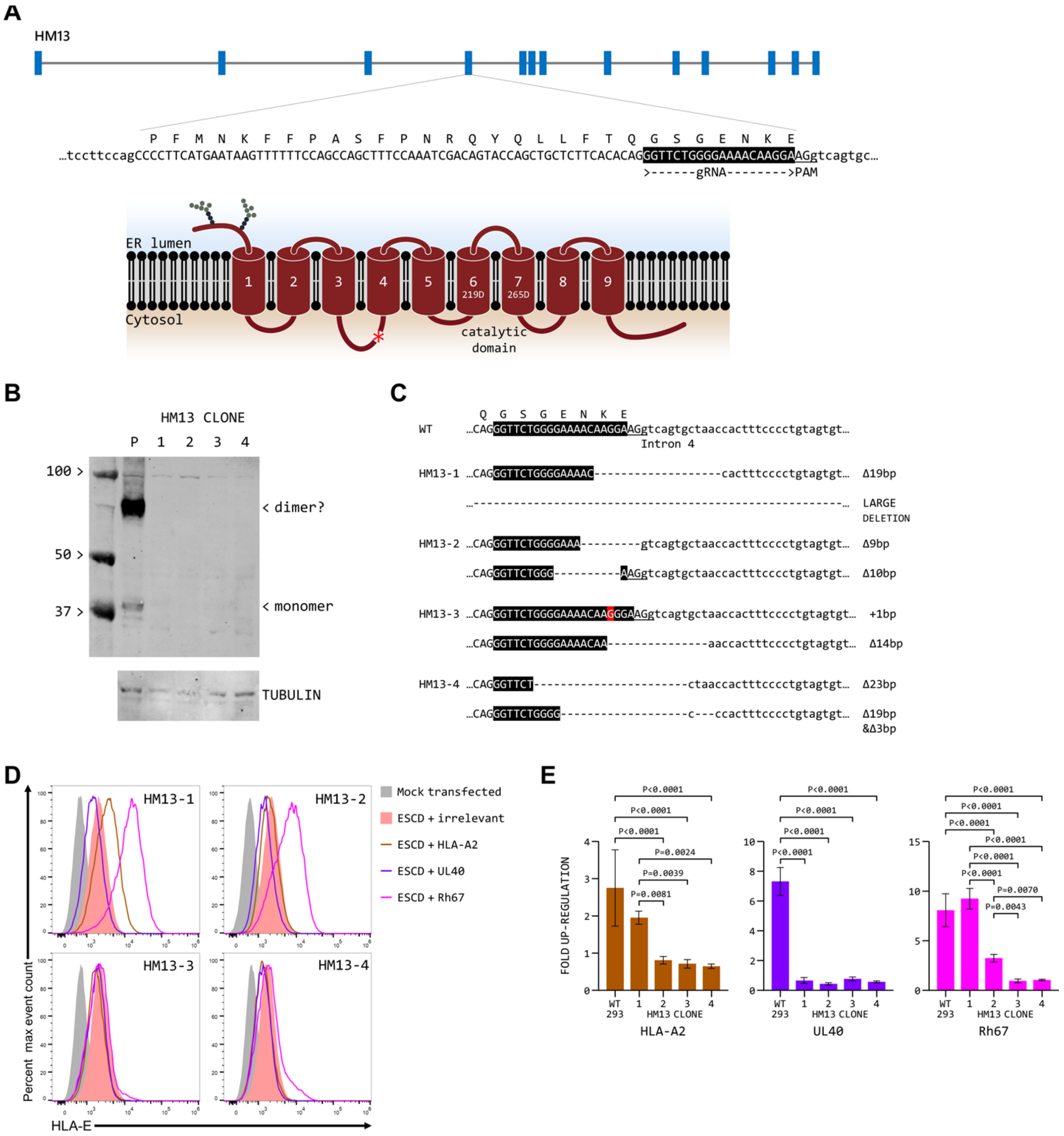

SUPPLEMENTARY FIGURE 5: Inactivation of Signal Peptide Peptidase (HM13) by CRISPR/Cas9

SUPPLEMENTARY FIGURE 5: Inactivation of Signal Peptide Peptidase (HM13) by CRISPR/Cas9

(A) Location of the gRNA target in exon 4 of the *HM13* gene, which encodes most of the longest cytoplasmic loop. Exon sequence is shown in uppercase and intron sequence in lowercase, with the sequence of the guide RNA indicated in white text on a black background and the adjacent PAM sequence (which overlaps the exon 4 donor splice site) underlined. The approximate location of the region of the protein encoded by the gRNA target sequence is indicated by the red asterisk on the schematic of the structure of the SPP protein (adapted from Supplementary Reference 2), which also has the locations of the two aspartate residues (positions 219 and 265) required for the catalytic activity of SPP indicated. **(B)** Western blot analysis with polyclonal anti-SPP of RIPA extracts of the parental 293T cells (P) or the four HM13 CRISPR/cas9 single cell clones. **(C)** Sequences of the genomic lesions in the alleles of *HM13* in the four single cell clones. NOTE: the primers used to amplify the region containing the CRISPR/Cas9 guide RNA target did not capture one of the *HM13* alleles in clone HM13-1 as this allele has a large deletion that removes part of intron 3 and the start of exon 4. **(D)** Representative HLA-E expression (of five independent replicates) in the four HM-13 CRISPR/Cas9 clones transfected with a plasmid expressing the HLA-E*01:03 SCD and plasmids expressing and irrelevant protein (HLA-B*57:01, filled blue histograms), HLA-A*02:01 (brown line), UL40 (purple line), or Rh67 (pink line). Mock transfected cells are shown as the filled grey histograms. **(E)** Fold up-regulation of expression of the HLA-E*01:03 SCD in the HM13 CRISPR/Cas9 clones, calculated as for Figure 1. Statistical analysis of the five independent replicates was by one way ANOVA with Tukey’s correction for multiple comparisons. Only statistically significant comparisons are indicated.

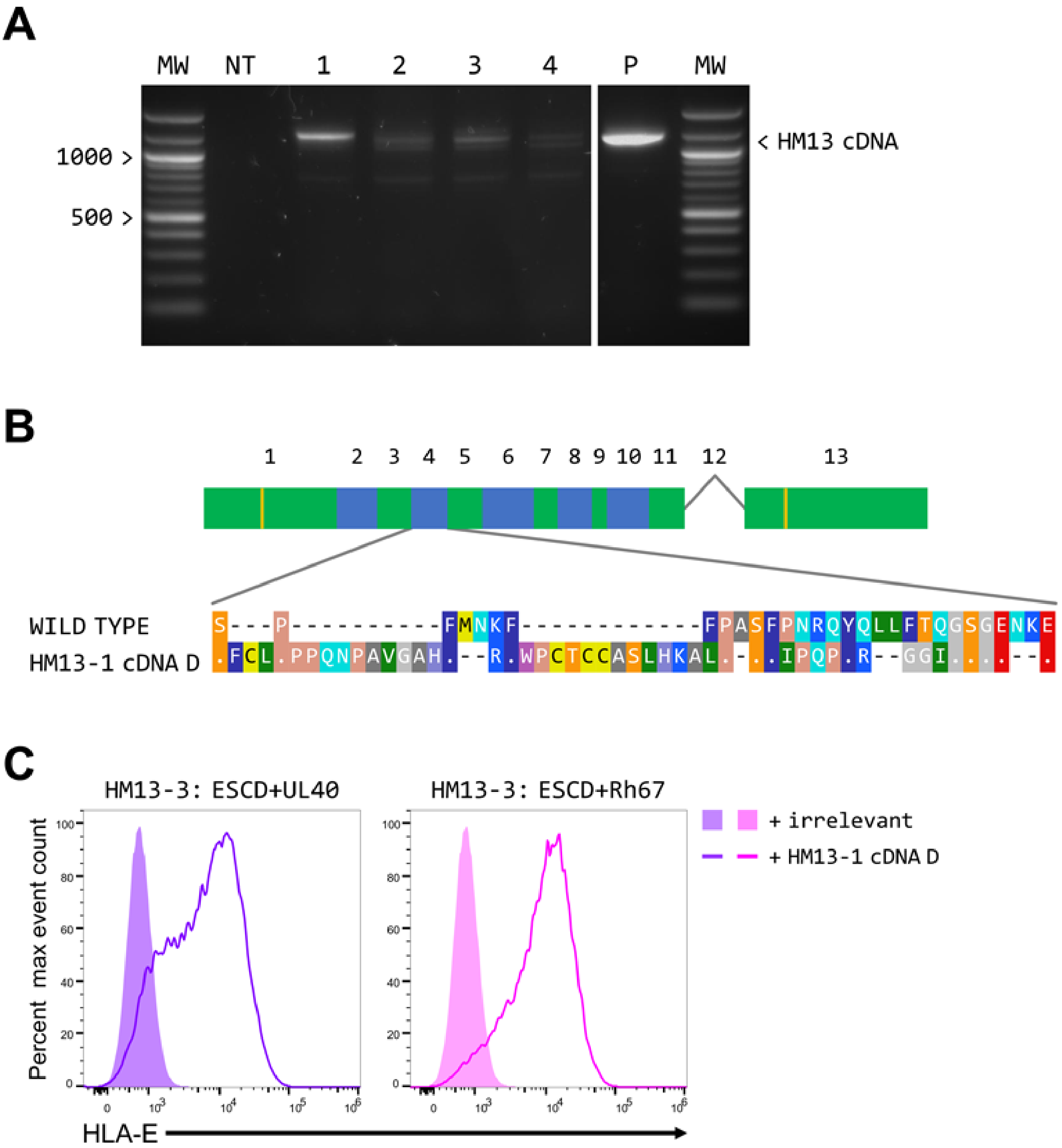

SUPPLEMENTARY FIGURE 6: Clone HM13-1 expresses a novel isoform of SPP that can act on both Rh67 and UL40

(A) Reverse Transcription PCR of *HM13* mRNA in the four HM13 CRISPR/Cas9 single cell clones, and the parental 293T cells (P). The lane marked NT was a no template control. Molecular weight (MW) markers (100 bp DNA Ladder, New England Biolabs) were run in the first and last lanes. Note that both images were from the same exposure of the same agarose gel, but have been shown separately as intervening lanes containing irrelevant samples have been removed. **(B)** Comparison of the sequence of exon 4 in SPP isoform 1 (which normally skips exon 12) with the exon 4 sequence in the novel isoform (cDNA D) predominantly expressed by one of the *HM13* alleles in clone HM13-1. The exon in the novel allele comprises sequence from intron 3 appended to the mutated end of exon 4. **(C)** Representative expression (of five independent replicates) of HLA-E in clone HM13-3 co-transfected with plasmids expressing the HLA-E*01:01 SCD and Rh67 (pink colour) or UL40 (purple colour) proteins, as well as plasmids expressing either an irrelevant protein (HLA-B*57:01, filled histograms) or HM13-1 cDNA D (coloured lines).

```
UL40 WT MNKFSNTRIGFTCAVMAPRTLVLTLGLLCMRIRSLLS
UL40 TT .............................T.T....
```

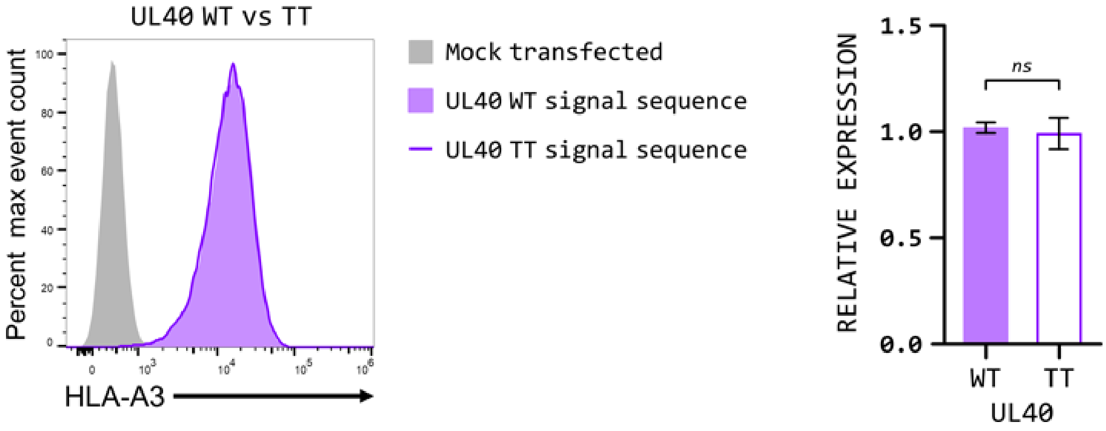

SUPPLEMENTARY FIGURE 7: The R31T+R33T mutation does not affect surface expression driven by the UL40 signal sequence

Representative expression (of five independent replicates) of an HLA-A*03:01 SCD with either the wild type UL40 signal sequence (filled purple histogram) or a version with the naturally-occurring arginine residues at positions 31 and 33 mutated to threonines (purple line), a mutation that was previously suggested to improve cleavage of the UL40 signal sequence by SPP (Lemberg and Martoglio 2002). Mock transfected cells are shown as the grey histogram. The graph shows expression ±SD (relative to that obtained with the HLA-A*02:01 signal sequence, as in Supplementary Figure 2) in the five replicates. Statistical analysis is from the same one way ANOVA with Tukey's correction for multiple comparisons shown in panel D of Supplementary Figure 2; *ns* denotes not significant.

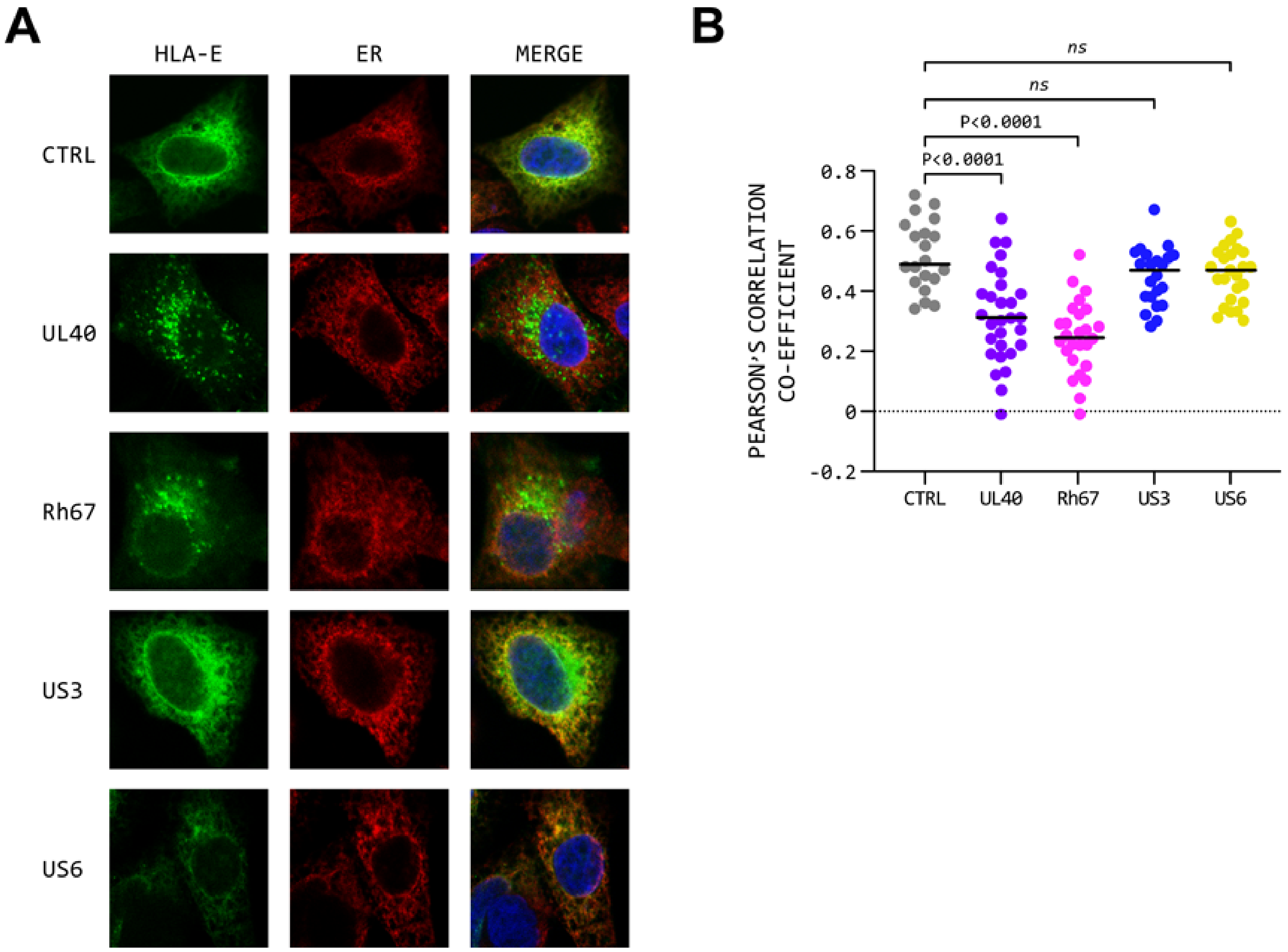

SUPPLEMENTARY FIGURE 8: UL40 and Rh67 reduce the amount of HLA-E in the ER

(A) Representative micrographs of HeLa cells stably expressing HLA-E*01:03 (HeLa.E), either untransfected (CTRL), or transiently transfected with plasmids expressing UL40. Rh67, US3, or US6. Endoplasmic Reticulum (ER) was visualised with an antibody specific for Calnexin. **(B)** Quantification of colocalization of HLA-E with ER in the transfected HeLa.E cells. Statistical analysis was by one-way ANOVA with Tukey's correction for multiple comparisons; *ns* denotes not significant.

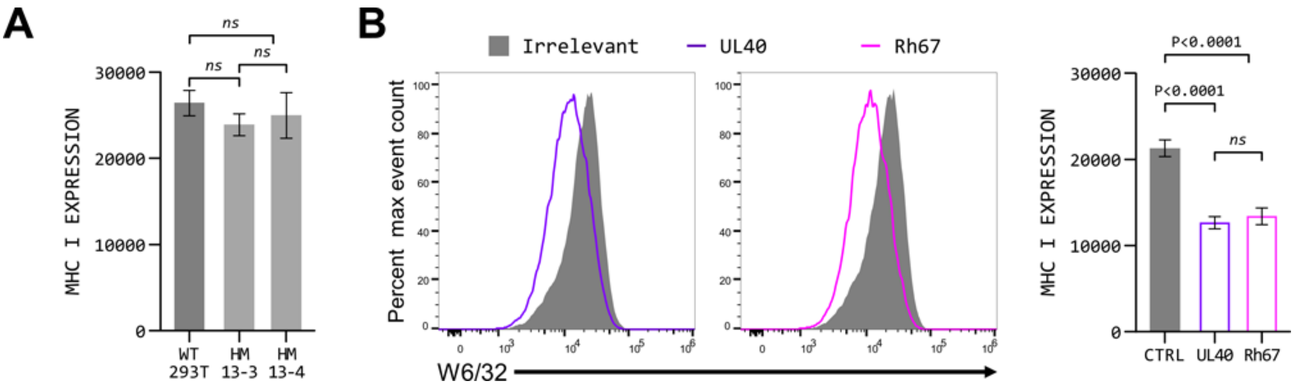

SUPPLEMENTARY FIGURE 9: Down-regulation of MHC class I expression in the SPP-deficient HM13-3 CIRSP/Cas9 clone

(A) MHC class I expression (±SD) of the two fully SPP-deficient HM13 CRISPR/Cas9 clones compared with that of the parental 293T cells for four independent replicates. **(B)** Representative cell surface MHC class I protein expression (of five independent replicates) for clone HM13-3 cells transfected with UL40 (left-hand histogram, purple line), or Rh67 (right-hand histogram, pink line). Cells transfected with a plasmid expressing an irrelevant protein (KIR2DS2) are shown as the filled dark grey histograms. In panel both panels, statistical analysis was performed using one-way ANOVA with Tukey's correction for multiple comparisons; *ns* denotes not significant.

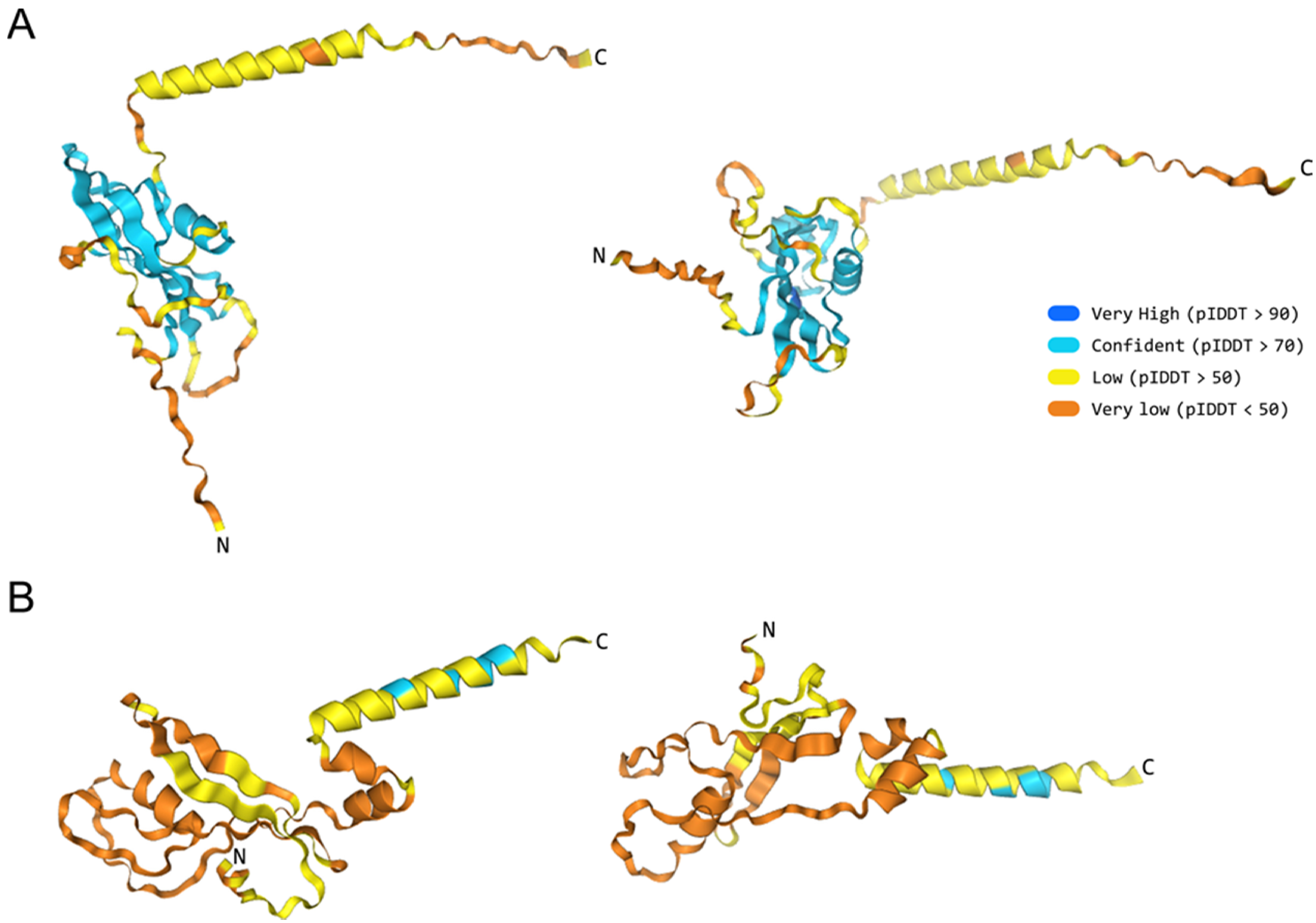

SUPPLEMENTARY FIGURE 10: AlphaFold3 predictions of the structures of the mature UL40 and Rh67 proteins

Two different views are shown of models for the mature proteins of UL40 (panel **A**) and Rh67 (panel **B**). Locations of the N and C termini are marked, and the models are coloured according to the per-atom confidence estimate (pLDDT, the scale boundaries shown in panel A).

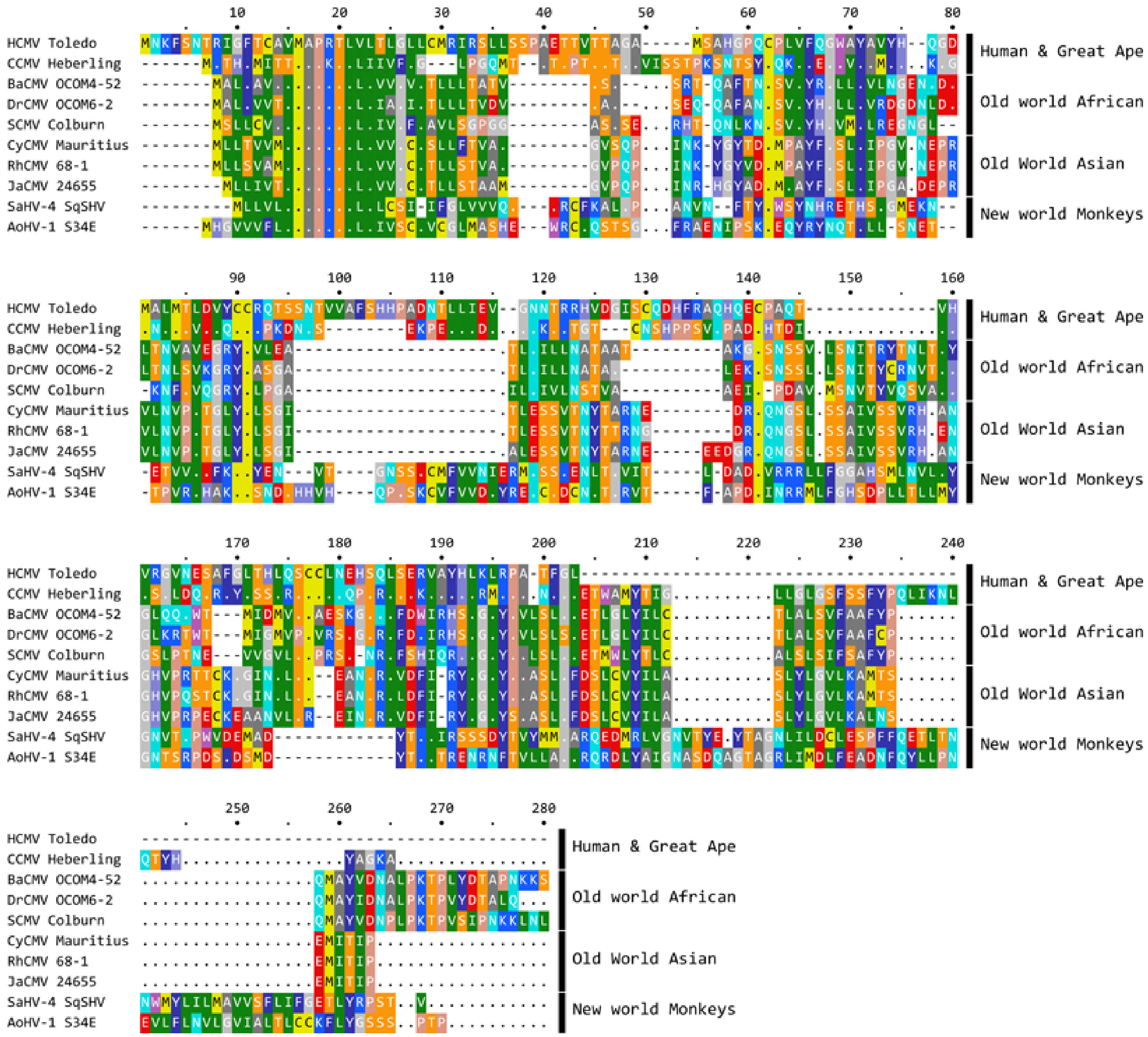

SUPPLEMENTARY FIGURE 11: Non-Human Primate UL40 candidates

ClustalΩ alignment (https://www.ebi.ac.uk/Tools/msa/clustalo/; Supplementary Reference 3) of the UL40 proteins encoded by 8 different NHP CMV genomes, along with HCMV UL40 (strain Toledo) and RhCMV (stain 68-1) Rh67. See Supplementary Table 9 for detail of the genomic sequences used. Abbreviations: CCMV, chimpanzee CMV; BaCMV, baboon CMV; DrCMV, Drill CMV; SCMV, Simian CMV; CyCMV, cynomolgus macaque CMV; JaCMV, Japanese macaque CMV; SaHV, Saimiriine betaherpesvirus; AoHV, Aotine betaherpesvirus 1.

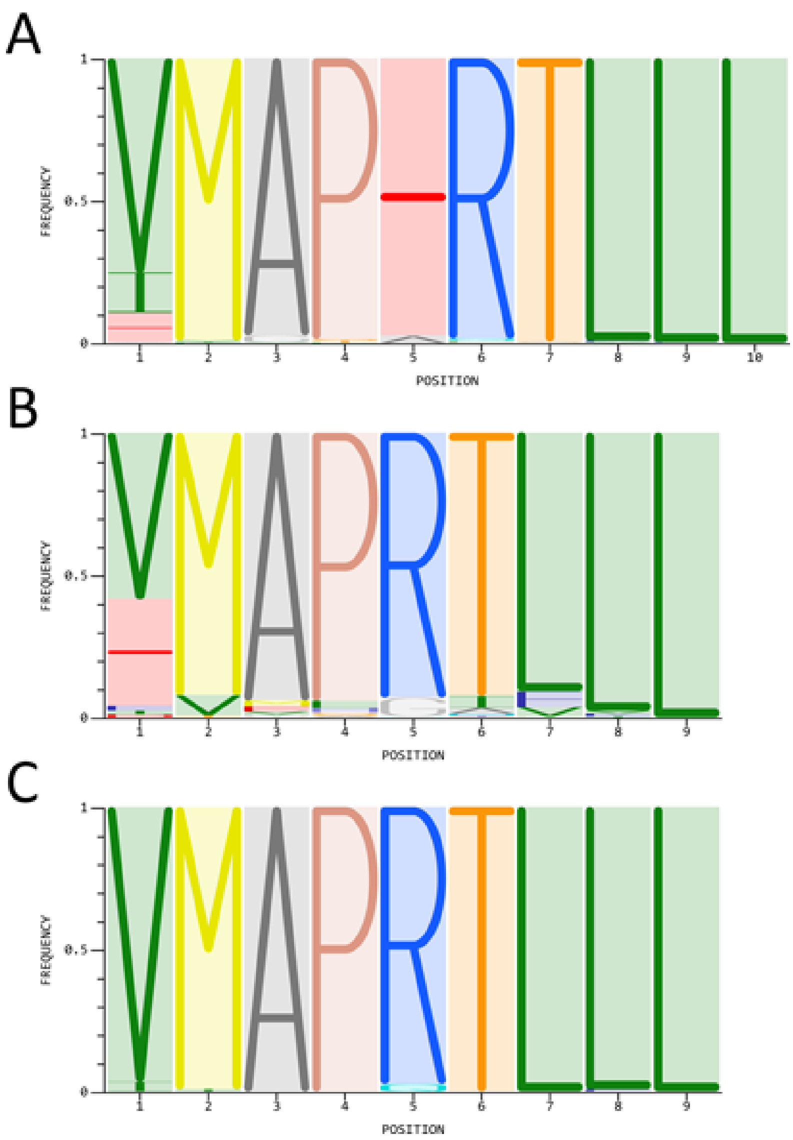

SUPPLEMENTARY FIGURE 12: Rhesus macaque VL9 sequence variation

Full length, functional sequences were extracted from release 3-12 of the IPD-MHC database (4) for Mamu-A (panel **A**, 535 sequences), Mamu-B (panel **B**, 816 sequences), and Mamu-AG (panel **C**, 151 sequences), and the relative frequencies of each amino acid at each position calculated. Note that the definition of full length used included those sequences that started with the methionine at position 2 of the VL9 peptide for Mamu-A (55 sequences, 10.3%) and Mamu-B (320 sequences, 39.2%), although it is likely that such sequences have been annotated with the wrong start codon. For Mamu-A, 13 sequences (2.4%) contain VL10 (VMAP**A**RTLLL) as the result of the presence of an additional alanine.

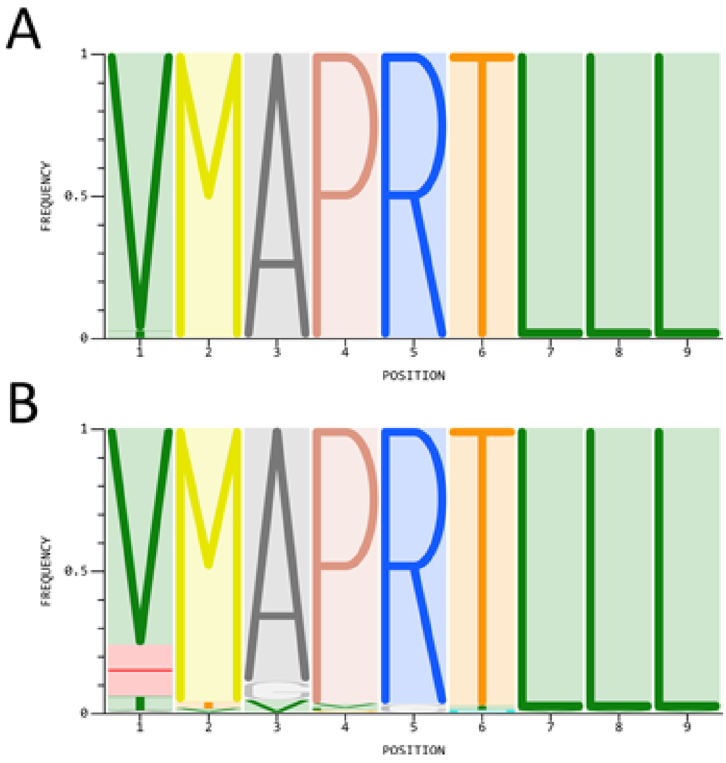

SUPPLEMENTARY FIGURE 13: Baboon VL9 sequence variation
Full length, functional sequences were extracted from release 3-12 of the IPD-MHC database (4) for Paan-A (panel **A**, 42 sequences), and Paan-B (panel **B**, 139 sequences), and the relative frequencies of each amino acid at each position calculated. Note that the definition of full length used included those sequences that started with the methionine at position 2 of the VL9 peptide for Paan-A (24 sequences, 17.3%), although it is likely that such sequences have been annotated with the wrong start codon.

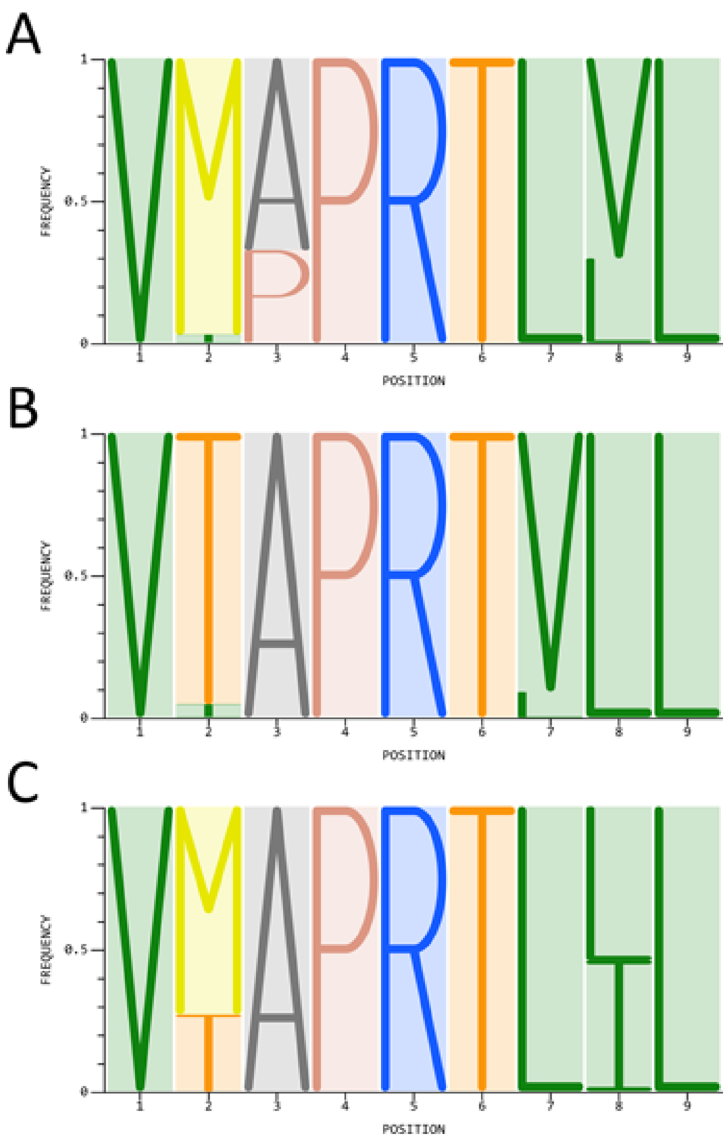

SUPPLEMENTARY FIGURE 14: Chimpanzee VL9 sequence variation

Full length, functional sequences were extracted from release 3-12 of the IPD-MHC database (4) for Patr-A (panel **A**, 37 sequences), Patr-B (panel **B**, 67 sequences), and Patr-C (panel **C**, 37 sequences), and the relative frequencies of each amino acid at each position calculated.

SUPPLEMENTARY TABLE 1: EXPRESSION CONSTRUCT PRIMERS

CONSTRUCT		PRIMER	SEQUENCE (5’-3')
PEPTIDE EXPRESSION	MVL9	FORWARD	AGCTTGCCACCATGGTGATGGCCCCTAGAACCCTGCTGCTTTGAA
		REVERSE	CCGGTTCAAAGCAGCAGGGTTCTAGGGGCCATCACCATGGTGGCA
	ESS-MVL9	FORWARD	GCAATGGTGATGGCCCCTAGAACCCTGCTGCTTTGAA
		REVERSE	CCGGTTCAAAGCAGCAGGGTTCTAGGGGCCATCACCATTGC
UL40	WT	FORWARD	TCAAGCTTGCCACCATGAACAAATTCAGCAACACTCGTATC
		REVERSE	GTGGATCCGCCTTTTTCAAGGCGTAGTGATGATCG
	R31T+R33T	FORWARD	CTCCTGTGTATGACGATCACGAGTTTATTGT
		REVERSE	GACAATAAACTCGTGATCGTCATACACAGGAG
	ΔN	FORWARD	TCAAGCTTGCCACCATGCGCGTTATGGCTCCGCGGACTTTAGTTCTGACGCTTGGAC
		REVERSE	AS WT
	ESS	FORWARD	CGTGCGCAATGAACAAATTCAGCAACACTCGTATC
		REVERSE	AS WT
Rh67	WT	FORWARD	TCAAGCTTGCCACCATGCTGCTCAGCGTGGCGATGGTGATGG
		REVERSE	GTGGATCCGGAATGGTTATCATTTCAGAAGTCATC
	ΔN	FORWARD	TCAAGCTTGCCACCATGCGCGTGATGGCTCCTAGGACTCTGCTTCTGGTGGTGG
		REVERSE	AS WT
	ESS	FORWARD	CTTGCGCAGTACCGCAGCCGATAAATAAATAC
		REVERSE	AS WT
UL40-A2 or Rh67-A2 hybrid signal sequences	UVA	FORWARD	TCAAGCTTGCCACCATGAACAAATTCAGCAACACTCGTATCGGCTTCACTTGCGC-GGTCATGGCGCCCCGAACCCTCGTCC
		REVERSE	GTACCGGTGGATCCACTTTACAAGCTGTGAGAGA
	RVA	FORWARD	TCAAGCTTGCCACCATGCTGCTCAGCGTGGCGATGGTCATGGCGCCCCGAACCCTCGTCC
		REVERSE	AS UVA
HM13 (SPP)	WT	FORWARD	CGGCTAGCGCCACCATGGACTCGGCCCTCAGCGATCCGCA
		REVERSE	GAGCGGCCGCTCATTTCTCTTTCTTCTCCAGCCCCTTCG
	D219A	FORWARD	CTCTTCATCTACGCTGTCTTCTGGGTATTTGG
		REVERSE	CAAATACCCAGAAGACAGCGTAGATGAAGAG

NOTE: The locations of mutations and restriction sites (full or partial) used for cloning are underlined

SUPPLEMENTARY TABLE 2:
CRISPR/CAS9 GUIDE RNA TARGETS

GENE	SEQUENCE
TAP1	5'-GAAAGACACTCAACCAGAAGG-3'
TAP2	5'-GTAGTCTCCTGGAAGAAACCG-3'
HM13	5'-GGTTCTGGGGAAAACAAGGA-3'

NOTE: Additional guanosines were added to the start of the *TAP1* and *TAP2* gRNAs to ensure efficient transcription

SUPPLEMENTARY TABLE 3: GENOMIC PCR PRIMERS

GENE	FORWARD PRIMER	REVERSE PRIMER
TAP1	5'-TGGTCCATGTTCCCAGGTTGCTC-3'	5'-CCTGAGAGGCAAAGGAAGGCCC-3'
TAP2	5'-CGCTGGGACAGAAGCAAGCA-3'	5'-ACAGCCCCACCACTTTCACCA-3'
HM13	5'-GCTCTGTTTGCCGACTTGCT-3'	5'-CCCAGGATCTCCACACTTCCC-3'

SUPPLEMENTARY TABLE 4:
GENETIC LESIONS OBSERVED IN THE ALLELES OF THE TAP1 CRISPR/CAS9 SINGLE CELL CLONES

CLONE	ALLELE 1	ALLELE 2
TAP1-1	Δ1bp: Premature stop in exon 6	Δ4bp: Premature stop in exon 6
TAP1-2	Δ3bp: 1 amino acid deleted	Δ4bp: Premature stop in exon 6
TAP1-3	Δ2bp: Premature stop in exon 5	Δ15bp: 5 amino acids deleted
TAP1-4	+1bp: Premature stop in exon 5	Δ11bp: Premature stop in exon 5

SUPPLEMENTARY TABLE 5:
GENETIC LESIONS OBSERVED IN THE ALLELES OF THE TAP2 CRISPR/CAS9 SINGLE CELL CLONES

CLONE	ALLELE 1	ALLELE 2
TAP2-1	UNMUTATED	+1bp: premature stop in exon 4
TAP2-2	+1bp: Premature stop in exon 4	+6bp: 2 amino acids added
TAP2-3	Δ6bp: 2 amino acids deleted	Δ6bp: 2 amino acids deleted
TAP2-4	+1bp: Premature stop in exon 4	Δ6bp: 2 amino acids deleted

SUPPLEMENTARY TABLE 6:
GENETIC LESIONS OBSERVED IN THE ALLELES OF THE HM13 CRISPR/CAS9 SINGLE CELL CLONES

CLONE	ALLELE 1	ALLELE 2
HM13-1	Δ19bp: exon 4 splice site removed	LARGE DELETION[1]
HM13-2	Δ9bp: 3 amino acids deleted[2]	Δ10bp: Premature stop in exon 5
HM13-3	+1bp: premature stop in exon 5	Δ14bp: Exon 4 splice site removed
HM13-4	Δ19bp: exon 4 splice site removed	Δ23bp & Δ3bp: Exon 4 splice site removed

NOTES:
1. The full extent of the deletion has not been determined, but it extends upstream of the target sequence into intron 3.
2. The three amino acids deleted are not contiguous: …GSGENKEEII… becomes …GSGEKII...

SUPPLEMENTARY TABLE 7:
5' SPLICE SITE PREDICTION FOR EXON 4 OF THE *HM13* GENE IN THE PARENTAL CELLS AND CLONE HM13-2

SPLICE SITE	SEQUENCE	MaxENT SCAN[1]	BDGP SP[2]
Optimal 5' splice site	…CAGgtaagt…	10.86	N/A[3]
Exon 4 *HM13* WT	…AAGgtcagt…	8.68	0.93
Exon 4 *HM13* clone 2	…AAAgtcagt…	1.98	NOT PREDICTED

NOTES:
1. MaxENT SCAN: http://hollywood.mit.edu/burgelab/maxent/Xmaxentscan_scoreseq.html
2. Berkley Drosophila Genome Project Splicing Prediction tool: https://www.fruitfly.org/cgi-bin/seq_tools/splice.pl
3. N/A = not applicable (splice sites predicted in situ, not with short sequences)

SUPPLEMENTARY TABLE 8: HM13 cDNA SPECIES IDENTIFIED IN CLONE HM13-1

cDNA	COUNT	SEQUENCE	ALLELE	PROTEIN
A	1/19	Exon 3 extended by 98bp, exons 4–6 skipped, premature stop codon in the extended exon 3 sequence	1	Non-functional
B	2/19	Exon 4 skipped, premature stop codon in exon 5	1	Non-functional
C	2/19	Exons 3–6 skipped, premature stop codon in exon 12	1	Non-functional
D	12/19	Exon 4 replaced by a novel (in frame) exon comprised of 136bp from intron 3, followed by GG, then the last 5bp of exon 4.	2	Functional?
E	1/19	As D but with exon 11 skipped, premature stop codon in exon 12	2	Cell surface?
F	1/19	As D but with exon 3 extended by 98bp (as in A), premature stop codon in the extended exon 3 sequence	2	Non-functional

SUPPLEMENTARY TABLE 9: NON-HUMAN PRIMATE CMV PROTEINS CONTAINING VL9 OR VL9-LIKE PEPTIDES

SPECIES	GENBANK	SEQUENCE	GENE	PEPTIDE
Pan troglodytes (Chimpanzee)	AF480884	Panine herpesvirus 2 strain Heberling, complete genome	UL40	TMAPKTLLI
	MZ151943	Panine betaherpesvirus 2 strain Heberling clone CCMV BAC-Phan9, complete sequence	UL40	TMAPKTLLI
Papio spp. (Baboon)	MT157321	Baboon cytomegalovirus isolate 31282, complete genome (*Papio anubis*)	UL40	VMAPRTLLL
	MT157322	Baboon cytomegalovirus isolate 34826, complete genome (*Papio hamadryas*)	UL40	VMAPTTLLL
	KR351281	Papiine betaherpesvirus 4 isolate OCOM4-52, complete genome (*Papio ursinus*)	n/a	VMAPTTLLL
Mandrillus leucophaeus (Drill)	KR297253	*Mandrillus leucophaeus* cytomegalovirus isolate OCOM6-2, complete genome	n/a	VMAPRTLLL
Cercopithecus aethiops (African Green Monkey)	FJ483969	Cercopithecine herpesvirus 5 strain Colburn, complete genome	UL40	VMAPRTLLL
	FJ483968	Cercopithecine herpesvirus 5 strain 2715, complete genome	UL40	VMAPRTLLL
	U27469	Stealth virus 1 strain ATCC VR-2343	n/a	VMAPRTLLL
Macaca fascicularis (Cynomolgus Macaque)	JN227533	Cynomolgus macaque cytomegalovirus strain Ottawa, complete genome	Cy63	VMAPRTLLL
	KP796148	Cynomolgus macaque cytomegalovirus strain Mauritius, complete genome	Cy63	VMAPRTLLL
	KX689263	Cynomolgus cytomegalovirus isolate 31906, complete genome	Cy67	VMAPRTLLL
	KX689264	Cynomolgus cytomegalovirus isolate 31907, complete genome	Cy67	VMAPRTLLL
	KX689265	Cynomolgus cytomegalovirus isolate 31908, complete genome	Cy67	VMAPRTLLL
	KX689266	Cynomolgus cytomegalovirus isolate 31907, complete genome	Cy67	VMAPRTLLL
	MT157323	Cynomolgus cytomegalovirus strain 31709, complete genome	Cy67	VMAPRTLLL
Macaca fuscata (Japanese Macaque)	MT157324	Japanese cytomegalovirus strain 24655, complete genome	Ja67	VMAPRTLLL
Saimiri sciureus (Squirrel Monkey)	FJ483967	Saimiriine herpesvirus 4 strain SqSHV, complete genome	UL40	VMAPRTLLL
Aotus trivirgatus (Owl Monkey)	FJ483970	Aotine herpesvirus 1 strain S34E, complete genome	UL40	VMAPRTLLL

NOTES:

1. *n/a means the relevant gene was not annotated in the GenBank entry*
2. *This table does not include any of the known Rhesus CMV Rh67 sequences*
3. *An alignment of representative sequences for each species is shown in Supplementary Figure 11*

SUPPLEMENTARY REFERENCES

1. Lehnert E, Tampe R. 2017. Structure and Dynamics of Antigenic Peptides in Complex with TAP. Front Immunol 8:10.

2. Voss M, Schroder B, Fluhrer R. 2013. Mechanism, specificity, and physiology of signal peptide peptidase (SPP) and SPP-like proteases. Biochim Biophys Acta 1828:2828-39.

3. Madeira F, Park YM, Lee J, Buso N, Gur T, Madhusoodanan N, Basutkar P, Tivey ARN, Potter SC, Finn RD, Lopez R. 2019. The EMBL-EBI search and sequence analysis tools APIs in 2019. Nucleic Acids Res 47: W636-W641.

4 Maccari G, Robinson J, Ballingall K, Guethlein LA, Grimholt U, Kaufman J, Ho CS, De Groot NG, Flicek P, Bontrop RE, Hammond JA and Marsh SGE. 2017. IPD-MHC 2.0: an improved inter-species database for the study of the major histocompatibility complex. Nucleic Acids Res. 45: D860-D864
